# A genome-wide cross-trait analysis identifies shared loci and causal relationships of type 2 diabetes and glycaemic traits with polycystic ovary syndrome

**DOI:** 10.1007/s00125-022-05746-x

**Published:** 2022-06-30

**Authors:** Qianwen Liu, Bowen Tang, Zhaozhong Zhu, Peter Kraft, Qiaolin Deng, Elisabet Stener-Victorin, Xia Jiang

**Affiliations:** 1 Department of Clinical Neuroscience, Karolinska Institutet, Solna, Stockholm, Sweden; 2 Department of Medical Epidemiology and Biostatistics, Karolinska Institutet, Solna, Stockholm, Sweden; 3 Department of Emergency Medicine, Massachusetts General Hospital, Harvard Medical School, Boston, MA USA; 4 Department of Biostatistics, Harvard T.H. Chan School of Public Health, Boston, MA USA; 5 Department of Epidemiology, Harvard T.H. Chan School of Public Health, Boston, MA USA; 6 Department of Physiology and Pharmacology, Karolinska Institutet, Solna, Stockholm, Sweden

**Keywords:** Genome-wide cross-trait analysis, Insulin resistance, Mendelian randomisation, Polycystic ovary syndrome, Type 2 diabetes

## Abstract

**Aims/hypothesis:**

The link underlying abnormal glucose metabolism, type 2 diabetes and polycystic ovary syndrome (PCOS) that is independent of BMI remains unclear in observational studies. We aimed to clarify this association using a genome-wide cross-trait approach.

**Methods:**

Summary statistics from the hitherto largest genome-wide association studies conducted for type 2 diabetes, type 2 diabetes mellitus adjusted for BMI (T2DM_adj_BMI), fasting glucose, fasting insulin, 2h glucose after an oral glucose challenge (all adjusted for BMI), HbA_1c_ and PCOS, all in populations of European ancestry, were used. We quantified overall and local genetic correlations, identified pleiotropic loci and expression–trait associations, and made causal inferences across traits.

**Results:**

A positive overall genetic correlation between type 2 diabetes and PCOS was observed, largely influenced by BMI (*r*_*g*_=0.31, *p*=1.63×10^–8^) but also independent of BMI (T2DM_adj_BMI–PCOS: *r*_*g*_=0.12, *p*=0.03). Sixteen pleiotropic loci affecting type 2 diabetes, glycaemic traits and PCOS were identified, suggesting mechanisms of association that are independent of BMI. Two shared expression–trait associations were found for type 2 diabetes/T2DM_adj_BMI and PCOS targeting tissues of the cardiovascular, exocrine/endocrine and digestive systems. A putative causal effect of fasting insulin adjusted for BMI and type 2 diabetes on PCOS was demonstrated.

**Conclusions/interpretation:**

We found a genetic link underlying type 2 diabetes, glycaemic traits and PCOS, driven by both biological pleiotropy and causal mediation, some of which is independent of BMI. Our findings highlight the importance of controlling fasting insulin levels to mitigate the risk of PCOS, as well as screening for and long-term monitoring of type 2 diabetes in all women with PCOS, irrespective of BMI.

**Graphical abstract:**

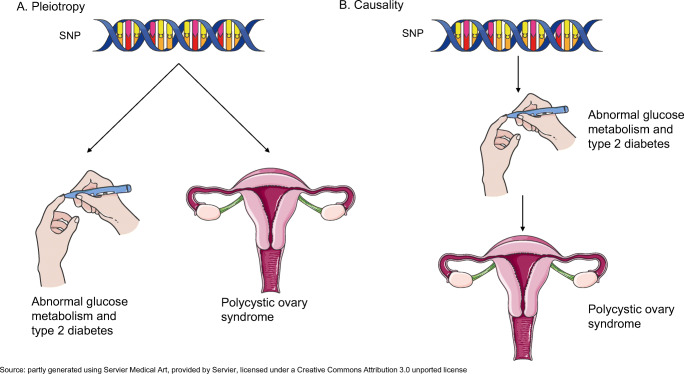

**Supplementary Information:**

The online version contains peer-reviewed but unedited supplementary material available at 10.1007/s00125-022-05746-x.

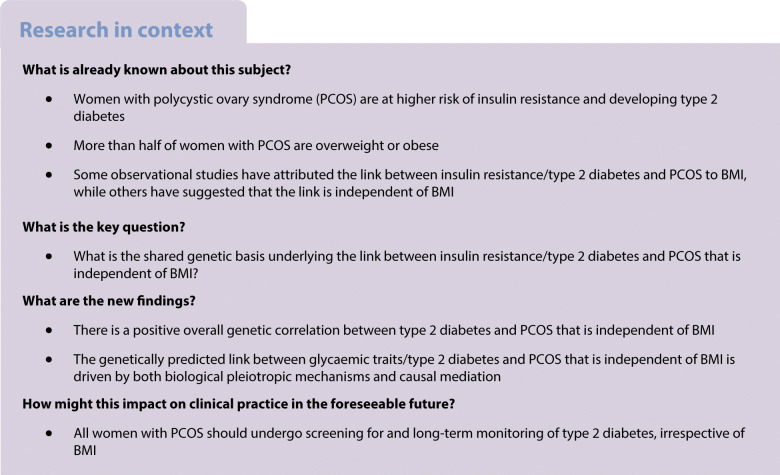



## Introduction

Polycystic ovary syndrome (PCOS) is the most common endocrine disorder affecting women of reproductive age. More than half of women with PCOS present with metabolic comorbidities, including obesity and insulin resistance, and women with PCOS are at a higher risk of developing type 2 diabetes [1–3], highlighting the importance of monitoring glucose metabolism for the prevention and management of PCOS.

Guidelines consistently recommend screening for type 2 diabetes in women with PCOS; however, a key question is whether screening should be offered to all patients or targeted only at those who are overweight or obese. Although insulin resistance and type 2 diabetes in PCOS are often believed to be attributed to BMI [4, 5], two large systematic reviews and meta-analyses suggest an effect that is independent of BMI [6, 7]. These inconclusive findings have posed challenges with regard to optimising clinical practice; however, there are methodological limitations of these studies because of the observational nature of conventional epidemiological investigations. The use of advanced study designs and unconfounded estimates of genetic associations could overcome such limitations and provide new insights into the underlying biology, which may aid clinical decision making.

Observational associations between two traits usually suggest shared environmental exposures and shared genetic components, because of genetic variants either having independent effects on both traits (horizontal pleiotropy or pleiotropy) or influencing one trait through their effect on the other (vertical pleiotropy or causality). Such shared genetic components can be dissected using a novel design named genome-wide cross-trait analysis [8, 9]. To the best of our knowledge, no such analysis has been conducted to comprehensively investigate the relationship between PCOS and its primary coexisting conditions, abnormal glycaemic metabolism and type 2 diabetes, taking BMI into consideration.

Therefore, in the current study we aimed to investigate the shared genetic contributions between type 2 diabetes, glycaemic traits and PCOS that are independent of BMI by conducting a comprehensive genetic analysis that leveraged the hitherto largest genome-wide association study (GWAS) summary statistics for each trait. We examined the role of type 2 diabetes, type 2 diabetes adjusted for BMI (T2DM_adj_BMI) [10], fasting glucose, fasting insulin, 2h glucose after an oral glucose challenge (all adjusted for BMI: FG_adj_BMI, FI_adj_BMI and 2hGlu_adj_BMI, respectively) and HbA_1c_ [11] in the development of PCOS [12] in people of European ancestry.

## Methods

### Study design

**Fig. 1:**
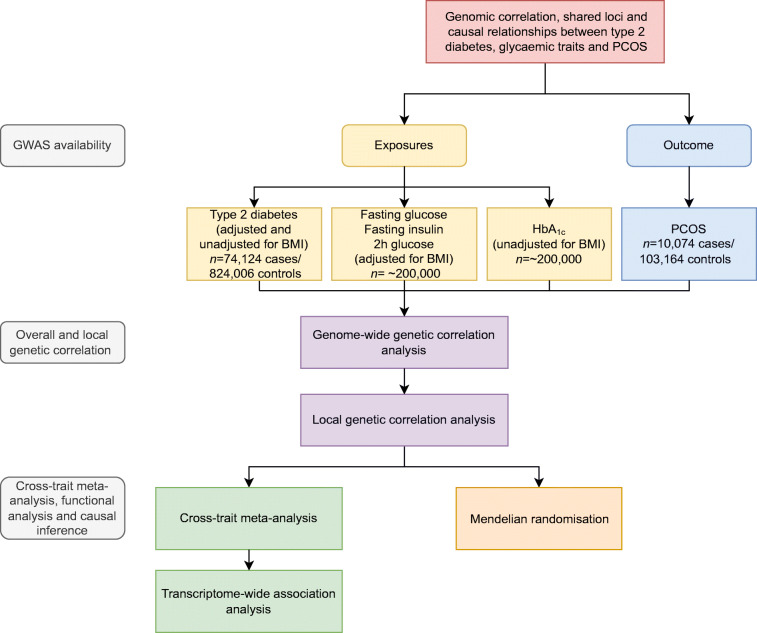
Illustration of the genome-wide cross-trait analysis design. We first quantified overall and local genetic correlation, then identified specific pleiotropic loci and detected expression–trait associations and finally inferred causal relationships. Genome-wide genetic correlation analysis: https://github.com/bulik/ldsc; local genetic correlation analysis: https://huwenboshi.github.io/hess/; cross-trait meta-analysis: http://hal.case.edu/~xxz10/zhu-web/; Mendelian randomisation: https://mrcieu.github.io/TwoSampleMR/; transcriptome-wide association analysis: http://gusevlab.org/projects/fusion/

An overview of the study design is shown in Fig. 1. We performed a genome-wide cross-trait analysis to quantify overall and local genetic correlation, identify pleiotropic loci, detect expression–trait associations and infer causal relationships.

### GWAS summary statistics for type 2 diabetes, glycaemic traits and PCOS

GWAS summary statistics for type 2 diabetes and T2DM_adj_BMI were obtained from the DIAbetes Genetics Replication And Meta-analysis (DIAGRAM) consortium; this dataset included 74,124 individuals with type 2 diabetes and 824,006 control participants from 32 European-ancestry GWASs [10]. Individuals with diabetes were identified based on WHO 1999 criteria (fasting plasma glucose ≥7.0 mmol/l or 2h plasma glucose ≥11.1 mmol/l) [13], HbA_1c_ ≥6.5%, casual glucose ≥11.1 mmol/l, use of diabetes medication or treatment for diabetes, medical records, ICD codes and self-report, either alone or in combination. The effect of each variant across all studies was combined using a fixed-effect meta-analysis of log ORs, yielding 231 type 2 diabetes-associated index SNPs of genome-wide significance (*p*<5×10^−8^). For T2DM_adj_BMI, the top associated SNPs were not reported by the original GWAS; we thus identified independent genome-wide significant (*p*<5×10^−8^) SNPs applying clumping at an *r*^2^<0.01 (see electronic supplementary material [ESM] Tables 1 and 2 for type 2 diabetes- and T2DM_adj_BMI-associated index SNPs, respectively).

GWAS summary statistics for glycaemic traits (FG_adj_BMI, FI_adj_BMI, HbA_1c_, 2hGlu_adj_BMI) were obtained from the Meta-Analyses of Glucose and Insulin-related traits Consortium (MAGIC); the dataset included 281,416 individuals without diabetes (70% European ancestry) [11]. For each glycaemic trait, adjustment was made for study-specific covariates and principal components. Independent genome-wide significant (*p*<5×10^−8^) SNPs in European ancestry were identified by this meta-GWAS, resulting in 85 FG_adj_BMI-associated index SNPs, 42 FI_adj_BMI-associated index SNPs, 86 HbA_1c_-associated index SNPs and 12 2hGlu_adj_BMI-associated index SNPs (ESM Tables 3–6, respectively).

GWAS summary statistics for PCOS, conducted through international collaborations, comprised 10,074 individuals with PCOS and 103,164 control participants of European ancestry. Diagnosis of PCOS was made based on the National Institutes of Health (NIH) or Rotterdam criteria, or by self-report [12]. In total, 14 independent genome-wide significant (*p*<5×10^–8^) SNPs were identified by meta-analysing the GWASs (ESM Table 7).

We extracted relevant information on each index SNP from each GWAS for Mendelian randomisation (MR) analysis and downloaded a full set of summary statistics for the other analyses. Detailed information on the characteristics of the GWAS data sources and the units for trait measurement are provided in ESM Table 8. All genetic data were aligned to the human reference genome build 37 (or hg19).

### Statistical analysis

#### Overall genetic correlation analysis

We performed a pairwise genetic correlation analysis using linkage disequilibrium score regression (LDSC), an algorithm that quantifies the average sharing of genetic effect across the whole genome between two traits unaffected by environmental confounders [14]. The final estimates ranged from –1 to 1, with –1 indicating a perfect negative genetic correlation and 1 indicating a perfect positive genetic correlation. We used pre-computed linkage disequilibrium (LD) scores obtained from ~1.2 million common SNPs in European ancestry represented in the HapMap3 reference panel, commonly believed to be of high imputation quality. A Bonferroni-corrected *p* value threshold of 0.05/6 was used to represent statistical significance.

#### Local genetic correlation analysis

Overall genetic correlation estimated by LDSC aggregates information across all variants in the genome. It is possible that, even though two traits show negligible overall genetic correlation, there are specific regions in the genome contributing to both traits. We therefore estimated the pairwise local genetic correlation using ρ-HESS (heritability estimation from summary statistics). This algorithm partitions the genome into 1703 prespecified LD-independent regions of 1.5 Mb and precisely quantifies genetic correlation restricted to each region [15]. A Bonferroni-corrected *p* value threshold of 0.05/1703 was used to represent statistical significance and *p*<0.05 was used as a suggestive significance threshold.

#### Cross-trait meta-analysis

Genetic correlation reflects either causality or pleiotropy. We therefore conducted a cross-trait meta-analysis at individual SNP level to identify pleiotropic loci shared between traits, using cross-phenotype association analysis (CPASSOC) [16]. CPASSOC integrates GWAS summary statistics from multiple correlated traits to detect variants associated with at least one trait, controlling for population structure or cryptic relatedness. The pairwise *S*_*Het*_ was calculated to combine summary statistics across traits. This test statistic (*S*_*Het*_) is an extension of *S*_*Hom*_ and is used more commonly in practice, showing improved power with heterogeneous genetic effects [16]. SNPs reaching genome-wide significance (*p*_CPASSOC_ <5×10^–8^) in paired traits and suggestive significance (*p*_single trait_ <1×10^–3^) in a single trait were considered significant pleiotropic SNPs.

We applied the PLINK clumping function to obtain independent SNPs (parameters: --clump-p1 5e-8 --clump-p2 1e-5 --clump-r2 0.2 --clump-kb 500) [17]. The Ensembl Variant Effect Predictor (VEP) was used for detailed functional annotation of the variants identified [18].

We categorised all CPASSOC-identified significant pleiotropic SNPs into one of four categories. The first category was ‘known’ shared SNPs that reached genome-wide significance in both single traits being analysed. These SNPs were identified as naturally shared SNPs even without CPASSOC testing. The second category was ‘single-trait-driven’ shared SNPs that reached genome-wide significance in either of the two single traits and in CPASSOC. The third category was shared SNPs that, despite not being driven by a single trait, were in LD with index SNPs previously identified in single-trait GWASs (LD *r*^2^> 0.2). Finally, the fourth category, novel SNPs, was prioritised by us and was of particular interest; novel SNPs were defined as shared SNPs that are neither driven by a single trait nor in LD with index SNPs identified in single-trait GWASs (LD *r*^2^< 0.2).

#### Transcriptome-wide association study

CPASSOC identifies genetic variants affecting multiple traits without considering gene expression or tissue specificity; however, many genetic variants influence complex traits by modulating gene expression levels [19]. To identify relevant genes whose expression patterns across tissues suggest a shared biological mechanism, we performed a transcriptome-wide association study (TWAS) using FUSION [19]. We first performed a single-trait TWAS leveraging the expression weights of 48 post-mortem tissues available at GTEx (version 7) and then intersected the single-trait TWAS results to examine if they were shared across traits. The false discovery rate (FDR) Benjamini–Hochberg correction (FDR <0.05) was used within each tissue to account for multiple comparisons.

#### Bidirectional Mendelian randomisation analysis

A two-sample MR analysis was conducted to evaluate causal associations. The inverse variance weighted (IVW) approach was used in the primary analysis assuming all instrumental variables (IVs) to be valid; the results would be biased even if only one IV was invalid [20]. We carried out a series of sensitivity analyses to determine the robustness of the results, including using a weighted median estimator method [21] and an MR-Egger regression [22], which gave consistent estimates under relaxed assumptions. We calculated the Cochran’s Q value to assess heterogeneity among individual IVs, with funnel plots created for visualisation. We used the MR pleiotropy residual sum and outlier (MR-PRESSO) framework as an additional check for pleiotropy and outliers. The global test detects pleiotropy among IVs and, when significant, the outlier test corrects for pleiotropy by outlier removal [23]. Both the MR-Egger regression and the IVW approach are based on the no measurement error (NOME) assumption, meaning that the variance of the SNP–exposure association is negligible, which can rarely be satisfied, leading to regression dilution bias in MR-Egger regression [24]. We evaluated the degree of such dilution using ${I}_{GX}^2$ and corrected for it using simulation extrapolation (SIMEX) [24]. To test if the causal estimate was driven by a single SNP, we performed a leave-one-out analysis in which each SNP was iteratively removed and the IVW approach was applied using the remaining SNPs. Steiger filtering was used to exclude all SNPs explaining more variance in the outcome than the exposure, after which the IVW method was repeated [25]. We then checked whether the results were consistent after excluding palindromic SNPs (A/T or G/C SNPs with the same pairs of letters on the forward and reverse strands).

Finally, to examine if genetic predisposition to PCOS influences type 2 diabetes and glycaemic traits, we performed a bidirectional MR analysis in which 14 genome-wide significant PCOS-associated SNPs were used as IVs [12]. 

A Bonferroni-corrected *p* value threshold of 0.05/6 and *p*<0.05 were used to represent statistical significance and suggestive significance, respectively.

All MR analyses were conducted in R version 4.1.2 (R Foundation for Statistical Computing, Vienna, Austria) using the packages ‘Two-SampleMR’, ‘SIMEX’ and ‘MR-PRESSO’.

## Results

### Overall and local genetic correlation

After correcting for multiple testing (*p*<0.05/6), we found a strong positive overall genetic correlation between type 2 diabetes and PCOS (*r*_*g*_=0.31, *p*=1.63×10^–8^) (Table 1). As BMI affects both traits in observational studies, we explored the genetic correlation between PCOS and T2DM_adj_BMI, in which the effect of BMI was controlled for. As expected, the positive genetic correlation was attenuated to less than half of its original value (*r*_*g*_=0.12, *p*=0.03), with suggestive significance indicating that the shared genetic basis was largely influenced by BMI but was also to a non-trivial extent independent of BMI. For glycaemic traits, we did not observe any significant overall genetic correlation with PCOS (FG_adj_BMI: *r*_*g*_=−0.04, *p*=0.54; FI_adj_BMI: *r*_*g*_=0.09, *p*=0.24; HbA_1c_: *r*_*g*_=0.13, *p*=0.06; 2hGlu_adj_BMI: *r*_*g*_=0.07, *p*=0.47).

**Table 1:** Genome-wide genetic correlation between type 2 diabetes/glycaemic traits and PCOS

Trait 1	Trait 2	*r*_*g*_	*r*_*g*__SE	*p* value
PCOS	T2DM	0.31	0.05	1.63×10^–8^
PCOS	T2DM_adj_BMI	0.12	0.06	0.03
PCOS	FG_adj_BMI	–0.04	0.07	0.54
PCOS	FI_adj_BMI	0.09	0.08	0.24
PCOS	HbA_1c_	0.13	0.07	0.06
PCOS	2hGlu_adj_BMI	0.07	0.10	0.47

*r*_g_, genetic correlation; T2DM, type 2 diabetes mellitus

After breaking down the genome into 1703 regions and correcting for multiple testing (*p*<0.05/1703), no significant local genetic correlation was identified between type 2 diabetes or glycaemic traits and PCOS (ESM Fig. 1). Suggestive significance (*p*<0.05) was observed for type 2 diabetes–PCOS at five genomic regions, for T2DM_adj_BMI–PCOS at six genomic regions, for FG_adj_BMI–PCOS at one genomic region and for HbA_1c_–PCOS at one genomic region (ESM Table 9).

### Cross-trait meta-analysis

CPASSOC identified 16 independent pleiotropic SNPs reaching genome-wide significance (*p*_CPASSOC_<5×10^–8^) in paired traits and suggestive significance (*p*_single trait_<1×10^–3^) in a single trait (Table 2). Notably, none of these 16 SNPs was previously reported to be associated with PCOS (0/16), while most of them were associated with at least one glycaemic trait or with type 2 diabetes (10/16).

**Table 2:** Cross-trait meta-analysis of type 2 diabetes/glycaemic traits and PCOS

SNP	CHR	BP	A1	A2	EAF	Beta_PCOS	Beta_trait	*p*_PCOS_	*p*_single trait_	*p*_CPASSOC_	Genes within clumping ranges
T2DM and PCOS											
rs8050136	16	53816275	A	C	0.41	0.15	0.12	4.50×10^–6^	7.60×10^–74^	1.95×10^–85^	*FTO*
rs9675376	18	57969244	A	G	0.3	0.13	0.04	2.40×10^–4^	3.90×10^–9^	4.47×10^–10^	
rs72753599	1	214180519	T	C	0.2	–0.14	–0.06	6.00×10^–4^	1.60×10^–12^	3.94×10^–13^	*PROX1*
rs10938398	4	45186139	A	G	0.42	0.11	0.04	8.80×10^–4^	4.90×10^–12^	1.02×10^–12^	
T2DM_adj_BMI and PCOS											
rs9930501	16	53830452	A	G	0.55	–0.14	–0.05	5.30×10^–6^	6.00×10^–13^	6.07×10^–15^	*FTO*
rs1509097^a^	2	165737931	T	C	0.5	–0.13	–0.04	5.20×10^–5^	5.30×10^–7^	3.34×10^–8^	*SLC38A11*
rs2238689	19	46178661	T	C	0.57	0.11	–0.07	5.40×10^–4^	1.40×10^–21^	9.67×10^–24^	*EML2*, *FBXO46*, *GIPR*, *MIR642A*, *MIR642B*, *QPCTL*, *SNRPD2*
rs72753599	1	214180519	T	C	0.2	–0.14	–0.07	6.00×10^–4^	1.60×10^–12^	6.26×10^–14^	*PROX1*
rs3934729^a^	3	123019763	T	C	0.61	–0.11	0.04	7.40×10^–4^	1.60×10^–7^	1.44×10^–8^	*ADCY5*, *SEC22A*
FG_adj_BMI and PCOS											
rs72753599	1	214180519	T	C	0.2	–0.14	–0.01	6.00×10^–4^	5.06×10^–12^	1.62×10^–12^	*PROX1*
rs9844212	3	123021870	C	G	0.59	–0.11	0.01	6.00×10^–4^	7.35×10^–13^	4.41×10^–13^	*ADCY5*, *SEC22A*
rs6485690	11	46798631	A	G	0.34	–0.11	0.01	9.40×10^–4^	4.60×10^–9^	1.06×10^–10^	*ARFGAP2*, *ARHGAP1*, *ATG13*, *C11orf49* (*CSTPP1*), *CKAP5*, *DDB2*, *F2*, *LRP4*, *LRP4-AS1*, *MIR5582*, *MIR6745*, *PACSIN3*, *SNORD67*, *ZNF408*
FI_adj_BMI and PCOS											
rs745379^a^	8	11615695	A	G	0.48	–0.12	–0.01	3.40×10^–4^	8.26×10^–6^	1.49×10^–9^	*C8orf49* (*LINC02905*), *FDFT1*, *GATA4*, *LINC00208*, *NEIL2*
rs3813583^a^	16	79755080	A	C	0.62	0.12	0.01	4.80×10^–4^	1.88×10^–4^	2.64×10^–8^	
rs4135247^a^	3	12396588	A	G	0.56	–0.11	–0.01	6.00×10^–4^	1.49×10^–6^	6.02×10^–9^	*PPARG*
HbA_1c_ and PCOS											
rs8047587^a^	16	53798622	T	G	0.45	0.14	0.01	6.80×10^–6^	1.87×10^–5^	2.87×10^–10^	*FTO*
rs1265564	12	111708458	A	C	0.58	0.13	0.01	1.60×10^–4^	5.62×10^–12^	2.91×10^–13^	*ACAD10*, *ATXN2*, *BRAP*, *CUX2*, *FAM109A* (*PHETA1*), *MIR6760*, *SH2B3*
rs2238689^a^	19	46178661	T	C	0.57	0.11	–0.01	5.40×10^–4^	1.45×10^–6^	1.12×10^–8^	*GIPR*, *MIR642A*, *MIR642B*
rs4731113	7	123283949	T	C	0.97	0.31	0.02	8.00×10^–4^	4.90×10^–8^	3.28×10^–9^	*LMOD2*, *WASL*

SNPs with *p*_CPASSOC_ <5×10^–8^ and *p*_single trait_ <1×10^–3^ are presented

^a^Novel SNPs, defined as shared SNPs that are neither driven by a single trait nor in LD with index SNPs identified in single-trait GWASs (LD *r*^2^<0.2)

A1, effect allele; A2, alternative allele; Beta, effect allele beta coefficient; BP, physical position of SNP (base pairs); CHR, chromosome; EAF: effect allele frequency; *p*_CPASSOC_, *p* value for cross-phenotype association; *p*_PCOS_, *p* value for PCOS; *p*_single trait_, *p* value for each individual trait; T2DM, type 2 diabetes mellitus

Four SNPs were shared between type 2 diabetes and PCOS (rs8050136, rs9675376, rs72753599 and rs10938398). The most significant shared locus was rs8050136 (*p*_CPASSOC_=1.95×10^–85^) located near *FTO*, which was also shared by T2DM_adj_BMI-PCOS (sentinel SNP [the most significant SNP at the locus]: rs9930501, *p*_CPASSOC_=6.07×10^–15^) and HbA1c-PCOS (sentinel SNP: rs8047587, *p*_CPASSOC_=2.87×10^–10^). The second most significant locus was rs72753599 (*p*_CPASSOC_=3.94×10^–13^) near *PROX1*, a gene that was also shared by T2DM_adj_BMI-PCOS (sentinel SNP: rs72753599, *p*_CPASSOC_=6.26×10^–14^) and FG_adj_BMI−PCOS (sentinel SNP: rs72753599, *p*_CPASSOC_=1.62×10^–12^).

Five SNPs were shared between T2DM_adj_BMI and PCOS (rs1509097, rs2238689, and rs3934729, in addition to rs9930501 and rs72753599 mentioned in the previous paragraph). The most significant SNP (rs2238689, *p*_CPASSOC_=9.67×10^–24^) was located near *GIPR*.

For FG_adj_BMI and PCOS, the most significant shared locus (sentinel SNP: rs9844212, *p*_CPASSOC_=4.41×10^–13^) was near *ADCY5*. This locus was also shared by T2DM_adj_BMI and PCOS (sentinel SNP: rs3934729, *p*_CPASSOC_=1.44×10^–8^).

Three SNPs were shared between FI_adj_BMI and PCOS (rs745379, rs3813583 and rs4135247), among which the most significant (sentinel SNP: rs745379, *p*_CPASSOC_=1.49×10^–9^) was located near *GATA4*, a PCOS-associated gene [12] that also plays an essential role in pancreatic development [26].

Among the four SNPs shared by HbA_1c_ and PCOS (rs8047587, rs1265564, rs2238689 and rs4731113), the most significant (rs1265564, *p*_CPASSOC_=2.91×10^–13^) was near *CUX2*, a gene that is expressed in neural tissues and that has previously been reported to be associated with insulin-dependent diabetes. *CUX2* directly regulates the expression of a transcription factor for the insulin gene [27].

Detailed annotations of each variant are shown in ESM Table 10.

### Transcriptome-wide association studies

Accounting for multiple testing (FDR <0.05) and across all tissues, single-trait TWAS identified 21 genes that are significantly associated with PCOS (ESM Table 11); 20806 genes were found to be significantly associated with type 2 diabetes, 11446 genes for T2DM_adj_BMI, 4241 genes for FG_adj_BMI, 2693 genes for FI_adj_BMI, 5702 genes for HbA_1c_, and 157 genes for 2hGlu_adj_BMI (ESM Fig. 2). Intersecting the single-trait TWAS results across traits, we identified one gene, *ARL14EP*, expressed in multiple tissues of the cardiovascular system and exocrine/endocrine system, that is shared between type 2 diabetes and PCOS. When the effect of BMI was removed, we found a second gene, *SERPINB8*, expressed in stomach, that is shared between T2DM_adj_BMI and PCOS (Table 3).

**Table 3:** Significant genes shared between type 2 diabetes/glycaemic traits and PCOS identified from the TWAS using gene expression across 48 GTEx tissues

Trait pair	CHR	Gene	Trait				PCOS			
			BEST.GWAS.ID	Tissue	TWAS.Z	FDR	BEST.GWAS.ID	Tissue	TWAS.Z	FDR
T2DM and PCOS	11	*ARL14EP*	rs4922556	Adipose subcutaneous, adrenal gland, artery aorta, artery coronary, artery tibial, brain caudate basal ganglia, brain cerebellum, breast mammary tissue, colon sigmoid, colon transverse, oesophagus gastroesophageal junction, oesophagus mucosa, oesophagus muscularis, heart atrial appendage, heart left ventricle, lung, nerve tibial, ovary, pituitary, skin not sun exposed suprapubic, skin sun exposed lower leg, stomach, thyroid, vagina	–2.83 to –3.89	4.84×10^−2^ to 2.73×10^−3^	rs10835649	Artery aorta, artery tibial, thyroid	4.36 to 5.24	1.49×10^−3^ to 4.99×10^−2^
T2DM_adj_BMI and PCOS	11	*ARL14EP*	rs4922556	Artery aorta, thyroid	–3.16 to –3.14	3.52×10^−2^ to 3.71×10^−2^	rs10835649	Artery aorta, artery tibial, thyroid	4.36 to 5.24	1.49×10^−3^ to 4.99×10^−2^
	18	*SERPINB8*	rs4508511	Nerve tibial, stomach	3.08 to 3.53	1.12×10^−2^ to 4.55×10^−2^	rs2162352	Stomach	–4.17	4.48×10^−2^

BEST.GWAS.ID, rsID of the most significant GWAS SNP in locus; CHR, chromosome; T2DM, type 2 diabetes mellitus; TWAS.Z, TWAS Z-score

### Bidirectional Mendelian randomisation

**Fig. 2:**
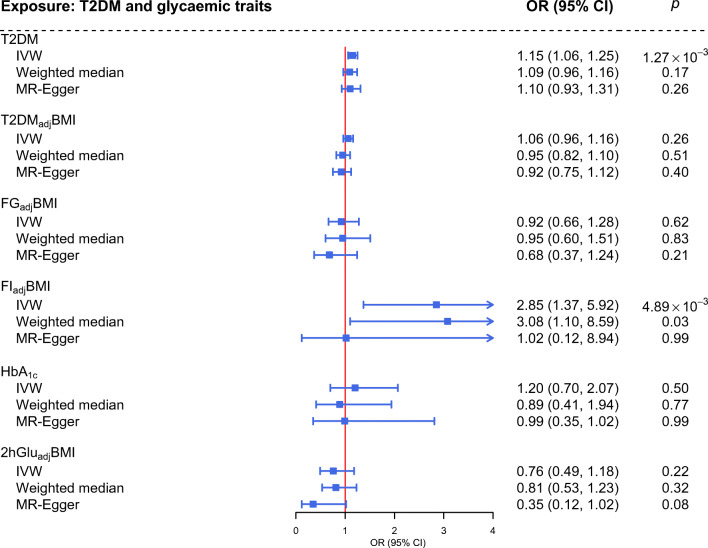
Estimates of the causal effects of genetically predicted type 2 diabetes and glycaemic traits on PCOS. The boxes denote the point estimates of the causal effects and the error bars denote the 95% CIs. The IVW approach was used in the primary analysis and the MR-Egger and weighted median approaches were used in sensitivity analyses. The ORs for PCOS were scaled to the per unit increase in log OR of type 2 diabetes and per unit increase in glycaemic traits. T2DM, type 2 diabetes mellitus

A significant causal effect of genetically predisposed type 2 diabetes on PCOS was observed using the IVW approach (OR 1.15, 95% CI 1.06, 1.25), which remined directionally consistent in MR-Egger regression (OR 1.10, 95% CI 0.93, 1.31) and using the weighted median approach (OR 1.09, 95% CI 0.96, 1.16) (Fig. 2). When the effect of BMI was removed, no causal association between T2DM_adj_BMI and PCOS was observed (IVW: OR 1.06, 95% CI 0.96, 1.16; MR-Egger: OR 0.92, 95% CI 0.75, 1.12; weighted median: OR 0.95, 95% CI 0.82, 1.10).

For glycaemic traits, a positive association between genetically predicted FI_adj_BMI and risk of PCOS was observed (IVW: OR 2.85, 95% CI 1.37, 5.92). The effect remained suggestively significant (*p*=0.03) using the weighted median approach (OR 3.08, 95% CI 1.10, 8.59). The MR-Egger regression yielded a directionally consistent estimate that was not significant (OR 1.02, 95% CI% 0.12, 8.94). No causal effect of any other glycaemic trait on PCOS was observed, as shown using the IVW approach (FG_adj_BMI: OR 0.92, 95% CI 0.66, 1.28; HbA1c: OR 1.20, 95% CI 0.70, 2.07; 2hGlu: OR 0.76, 95% CI 0.49, 1.18); the same results were found using the other two approaches (Fig. 2).

We observed directionally consistent results in the sensitivity analyses performed, corroborating the robustness of the findings (ESM Results, ESM Tables 12-16 and ESM Figs 3–7).

Finally, genetically predisposed PCOS did not seem to affect type 2 diabetes or any of the glycaemic traits, with all effects close to null in reverse MR analysis (ESM Fig. 8). All 14 SNPs explained more variance in PCOS than in type 2 diabetes or glycaemic traits, meaning that no SNPs were removed in Steiger filtering.

## Discussion

To the best of our knowledge, this is the first large-scale genome-wide cross-trait analysis investigating the genomic correlation, pleiotropic loci, expression–trait associations and causal relationships between type 2 diabetes or glycaemic traits and PCOS. We found a positive overall type 2 diabetes–PCOS genetic correlation, which was largely driven by, but was also independent of, BMI, indicating a shared genetic basis as a result of pleiotropy or causality. We next identified 16 pleiotropic SNPs that are shared across traits and two expression–trait associations tagging tissues of the cardiovascular, exocrine/endocrine and digestive systems, suggesting a common biology. We further demonstrated a putative causal role of genetically predicted type 2 diabetes and FI_adj_BMI in the development of PCOS, supporting a role of interventions on fasting insulin levels in the prevention of PCOS.

Our findings are largely in line with those from previous studies, yet extend these findings in several important ways. First, leveraging summary statistics from the hitherto largest GWASs, our study substantially improves the statistical power of genetic correlation analysis. Day et al identified a positive type 2 diabetes–PCOS genetic correlation [12] using a type 2 diabetes GWAS including 34,840 individuals with diabetes and 114,981 control participants, which we replicated using a sample size that was sixfold higher (using the most recently published GWAS involving 74,124 individuals with diabetes and 824,006 control participants). On the other hand, although a positive genetic correlation was revealed for FI_adj_BMI (96,496 individuals) and PCOS by the same authors [12], our analysis, with double the sample size (~200,000 individuals), did not support such a finding. Second, in MR analysis, incorporating additional IVs (42 vs 12 FI_adj_BMI SNPs) derived from large-scale GWASs substantially improves the strength of genetic instruments as well as both the accuracy and the precision of causal estimates. With the current sample size for the outcome of PCOS (*n=*113,238, 9% cases), and assuming that the phenotypic variance of the exposures explained by IVs is around 0.62% [11], we were able to detect an association of a 38% change in the risk of PCOS with FI_adj_BMI with 80% power. Third, while a previous MR analysis reported only a null PCOS–type 2 diabetes causal association [28], our bidirectional MR analysis, which took into consideration reverse causation, found a novel type 2 diabetes–PCOS causal association, suggesting that a genetic predisposition to type 2 diabetes plays an important role in PCOS development. A fourth advancement is the consideration of the effect of BMI. While previous observational studies have found inconsistent results on whether the link between type 2 diabetes/glycaemic traits and PCOS can be entirely attributed to BMI [4–7, 29], our findings support a pathogenesis pathway that is independent of BMI. BMI may not be sufficient at reflecting adiposity, yet the results from our previous investigation largely supported the role of BMI rather than fat distribution (waist-to-hip ratio with and without adjusting for BMI) in the development of PCOS [30]. Waist circumference (WC) [31], another and potentially better indicator of abdominal fat, was examined and a positive genetic correlation was identified with PCOS (*r*_*g*_=0.46, *p*=5.32×10^−11^). However, when the effect of BMI was removed, the prior positive WC–PCOS genetic correlation was attenuated to null (WC_adj_BMI: *r*_*g*_=0.08, *p*=0.19). This evidence collectively supports the role of BMI rather than fat distribution in the pathogenesis of PCOS, suggesting the adequacy and appropriateness of adjusting only for BMI in the current study. Results from multivariable MR adjusting for female adult BMI [32] consistently support a suggestive direct type 2 diabetes–PCOS causal association that is independent of BMI (IVW: OR 1.09, *p*=0.04).

In addition to the genetic correlations and causal relationships (type 2 diabetes–PCOS and/or FI_adj_BMI–PCOS) identified by our study, results from cross-trait meta-analysis suggest that the observational link may largely be explained by potential pleiotropic variants affecting both traits independently and by mechanisms that are independent of BMI. Here we highlight four novel SNPs (as defined in the Methods) with interesting findings. The first of these is rs3934729 located near *ADCY5*, a gene shared by T2DM_adj_BMI, FG_adj_BMI and PCOS and overlapping a suggestively significant T2DM_adj_BMI–PCOS local genetic correlation region chr3:11019665-13070799. Variation in *ADCY5* increases fasting glucose levels and type 2 diabetes risk through altered expression in beta cells and impaired glucose signalling [33] and has been found to decrease the disposition index (an indicator of insulin secretion capacity) in women with gestational diabetes after adjusting for BMI [34]. Variation in *ADCY5* has also been shown to affect ovarian morphological-related traits in bovines [35]. The second novel SNP, rs8047587, is located near *FTO*, a gene shared by HbA_1c_, T2DM_adj_BMI and PCOS. Candidate gene studies have suggested that *FTO* variation is associated with insulin resistance or hyperinsulinaemia in women with PCOS, independent of BMI [36]. The third novel SNP, rs2238689, shared by T2DM_adj_BMI, HbA_1c_ and PCOS, is located near *GIPR*, which encodes a G protein-coupled receptor for gastric inhibitory polypeptide expressed in the pituitary and ovaries. Variation in *GIPR* is known to lead to impaired glucose tolerance and type 2 diabetes through an impaired incretin (a gut-derived peptide hormone) effect [37]. Recent work has linked the amelioration of PCOS after weight-loss bariatric surgery to an improved gut hormonal milieu, highlighting the role of gut hormone receptor modulation in PCOS [38]. The fourth novel SNP, rs4135247, is shared by FI_adj_BMI and PCOS and is located near *PPARG*, a gene involved in the insulin signalling pathway in type 2 diabetes that has been found to be associated with PCOS susceptibility [39]. In addition to novel shared SNPs, we further highlight one single-trait-driven SNP of interest, rs72753599, located near *PROX1*, a gene known to alter beta cell insulin secretion [40], which was shared by type 2 diabetes, T2DM_adj_BMI, FG_adj_BMI and PCOS. At first glance, *PROX1* seems to play no major role in PCOS; however, a previous study found that *PROX1* affects the pathogenesis of PCOS through its involvement in lymphatic vasculature in the ovary [41]. Finally, a TWAS identified one gene shared by T2DM_adj_BMI and PCOS, suggesting potential shared biology through a protein encoded by *ARL14EP*. *ARL14EP* is expressed in the aorta, tibial artery, thyroid and ovary, among other tissues. *ARL14EP* encodes an effector protein that interacts with ADP-ribosylation factor-like 14, which may control the movement of MHC class II-containing vesicles, contributing to a PCOS diagnosis based on the NIH criteria, which presents the greatest risk for insulin resistance and other metabolic disorders [42]. All these findings suggest a biological mechanism that is independent of BMI. Further studies are needed to replicate and verify our findings. Another mechanism that may explain the higher risk of type 2 diabetes in PCOS is testosterone excess [43].

From translational and clinical perspectives, our findings clarify that both a shared genetic aetiology and causal effects explain the observational link between abnormal glucose metabolism and type 2 diabetes and PCOS, and deliver two messages that may inform clinical practice. First, findings of pleiotropic variants highlight a shared aetiology underlying glycaemic traits and type 2 diabetes and PCOS, in which women with PCOS are inherently at a higher risk of abnormal glucose metabolism and type 2 diabetes through pathways that are independent of BMI, supporting the need for long-term, regular monitoring of glycaemic status in these individuals. Second, findings of the FI_adj_BMI–PCOS and type 2 diabetes–PCOS (univariable MR and multivariable MR) causal associations suggest the importance of controlling fasting insulin levels to mitigate the risk of developing PCOS, irrespective of BMI. From a broader public health perspective, lifestyle interventions (e.g. exercise and diet modification) may improve glucose metabolism and decrease PCOS risk simultaneously.

We acknowledge a few limitations. First, because of limited data availability, we were unable to use sex-specific GWAS data on type 2 diabetes and glycaemic traits to match the data on the female-specific outcome PCOS at the time of conducting the analysis. However, sex heterogeneity did not seem to play a significant role when using female-specific type 2 diabetes GWASs, which are now available through the DIAGRAM consortium website (https://diagram-consortium.org/index.html; accessed 17 March 2022) (female *r*_*g*_=0.33, *p*=1.24×10^−7^; ESM Table 17, ESM Fig. 9). Although underpowered, female-specific MR analysis yielded directionally consistent findings (IVW: OR 1.08, *p*=0.24) to type 2 diabetes−PCOS MR findings using the sex-combined type 2 diabetes GWAS (OR 1.15, 95% CI 1.06, 1.25). Using sufficiently powered female-specific data could thus be a future direction for research. Second, PCOS encompasses genetically heterogeneous subtypes, as recently classified in an unsupervised clustering analysis [44]; however, we were unable to assess these subtypes because of limited data availability. Third, the generalisability of our findings was restricted to European ancestry populations. Fourth, although statistical power was greatly improved in our analysis compared with previous MR analyses, we acknowledge that the phenotypic variance explained by IVs for some traits remains modest. Therefore, studies with even greater statistical power are warranted. However, the instruments used were sufficiently strong, as reflected by the *F* statistics (ESM Table 8). Finally, nearly all the included exposure GWASs (except for HbA_1c_) were adjusted for BMI. Although this enables effects to be interrogated independently of BMI, it is also likely to introduce collider bias, which could violate the independence assumption (IVs are not associated with confounders) [45]. In the glycaemic GWAS study, Chen et al confirmed that collider bias influenced less than 2% of the glycaemic signals [11]; our MR results are also most likely to be unbiased, although they should be interpreted with caution.

To conclude, leveraging the hitherto largest genome-wide genetic data and advanced statistical genetics approaches, our study provides novel insights into the observational associations of type 2 diabetes and glycaemic traits with PCOS. Our findings suggest that such associations are driven in part by pleiotropic effects and in part by causal effects of a genetic predisposition to type 2 diabetes and of fasting insulin on the development of PCOS, which are independent of BMI.

## Supplementary Information

ESM (PDF 2785 kb)

## Data Availability

All GWAS summary statistics are publicly available.

## Abbreviations

2hGlu_adj_BMI: 2h glucose after an oral glucose challenge adjusted for BMI
CPASSOC: Cross-phenotype association analysis
FDR: False discovery rate (Benjamini–Hochberg correction)
FG_adj_BMI: Fasting glucose adjusted for BMI
FI_adj_BMI: Fasting insulin adjusted for BMI
GWAS: Genome-wide association study
IV: Instrumental variable
IVW: Inverse variance weighted
LD: Linkage disequilibrium
LDSC: Linkage disequilibrium score regression
MR: Mendelian randomisation
NOME: No measurement error
PCOS: Polycystic ovary syndrome
SIMEX: Simulation extrapolation
T2DM_adj_BMI: Type 2 diabetes mellitus adjusted for BMI
TWAS: Transcriptome-wide association study
WC: Waist circumference
