# Supplementary material for: A genome-wide cross-trait analysis identifies shared loci and causal relationships of type 2 diabetes and glycaemic traits with polycystic ovary syndrome
Source: Diabetologia. 2022 Jun 30;65(9):1483–94. doi: 10.1007/s00125-022-05746-x (PMC9345824; doi:10.1007/s00125-022-05746-x)
Supplement: Supplementary file 1 — (PDF 2785 kb) [file 125_2022_5746_MOESM1_ESM.pdf]

ESM Results: MR results from sensitivity analyses for causal effect of T2DM, glycaemic traits on PCOS.

We conducted several sensitivity analyses to assess the robustness of results against violation to MR assumptions. We did not observe any evidence of directional pleiotropy from the MR-Egger regression intercepts ($P_{\text{MR-Egger intercept}} > 0.05$) (**ESM Table 12).** The I^2_{GX} value assessing the degree of dilution bias from MR-Egger regression suggested a 27% dilution of the $FI_{adj}BMI$-PCOS estimate (I^2_{GX} =0.73) and a 24% dilution of the $2hGlu_{adj}BMI$-PCOS estimate (I^2_{GX}=0.76) due to violating the NOME assumption (**ESM Table 13**). Correcting for dilution bias, the estimates restored consistency with results from IVW (**ESM Table 12**), strengthening the significant $FI_{adj}BMI$-PCOS causal association. Despite Cochran's Q-values suggested substantial between-SNP heterogeneity in the analysis of type 2 diabetes mellitus, $T2DM_{adj}BMI$, and $2hGlu_{adj}BMI$ with PCOS (**ESM Table 14**), the insufficient asymmetry of funnel plots (**ESM Figs. 3-5**) supported a lack of directional pleiotropy. After excluding few outliers indicated by MR-PRESSO, the causal estimates showed consistency with the raw IVW findings (**ESM Table 15**). Results remained similar in leave-one-out analysis (**ESM Fig. 6**), suggesting that the overall causal effect estimates were not driven by outlying SNPs. All MR estimates after Steiger filtering remained directionally consistent with our main MR results (**ESM Table 16**). Similar results were observed when excluding palindromic SNPs (**ESM Fig. 7**).

ESM Table 1. Characteristics of genome-wide significant single nucleotide polymorphisms associated with T2DM.

Nearest gene	SNP	CHR	POS	A1	A2	EAF	Beta	SE	*p*-value
MACF1	rs3768321	1	40035928	T	G	0.20	0.086	0.005	2.60E-26
FAF1	rs58432198	1	51256091	C	T	0.88	0.068	0.009	2.10E-10
PATJ	rs12140153	1	62579891	G	T	0.91	0.068	0.009	1.30E-08
DENND2C	rs184660829	1	115144899	C	T	0.00	2.086	0.375	2.50E-08
PTGFRN	rs1127215	1	117532790	C	T	0.58	0.049	0.005	1.60E-13
NOTCH2	rs1493694	1	120526982	T	C	0.11	0.086	0.009	2.70E-16
FAM63A	rs145904381	1	151017991	T	C	0.99	0.174	0.029	2.60E-08
SEC16B	rs539515	1	177889025	C	A	0.20	0.049	0.010	1.60E-10
DSTYK	rs12048743	1	205114873	G	C	0.44	0.039	0.005	3.50E-09
SRGAP2	rs9430095	1	206593900	C	G	0.49	0.039	0.005	1.90E-08
PROX1	rs340874	1	214159256	C	T	0.56	0.068	0.005	1.60E-22
LYPLAL1	rs2820446	1	219748818	C	G	0.71	0.058	0.005	3.30E-16
ABCB10	rs348330	1	229672955	G	A	0.36	0.049	0.010	2.70E-14
GNG4	rs291367	1	235690800	G	A	0.63	0.039	0.010	4.70E-10
TMEM18	rs62107261	2	422144	T	C	0.95	0.113	0.013	3.80E-12
FAM49A	rs11680058	2	16574669	A	G	0.86	0.058	0.010	1.40E-08
DTNB	rs17802463	2	25643221	G	T	0.73	0.039	0.005	2.90E-08
GCKR	rs1260326	2	27730940	C	T	0.61	0.068	0.005	6.50E-25
THADA	rs80147536	2	43698028	A	T	0.90	0.122	0.013	2.70E-29
BNIPL	rs6545714	2	59307725	G	A	0.39	0.039	0.005	8.90E-09
BCL11A	rs243024	2	60583665	A	G	0.46	0.058	0.005	2.50E-20
CEP68	rs2249105	2	65287896	A	G	0.63	0.095	0.014	2.20E-14
DDX18	rs562386202	2	118071061	G	A	0.00	1.163	0.213	4.20E-08
GLI2	rs11688682	2	121347612	G	C	0.73	0.049	0.005	4.20E-09
PABPC1P2	rs35999103	2	147861633	T	C	0.15	0.049	0.010	9.70E-09
CYTIP	rs13426680	2	158339550	A	G	0.94	0.086	0.009	6.70E-10
RBMS1	rs3772071	2	161135544	T	C	0.71	0.049	0.005	1.20E-11
GRB14/COBLL1	rs10195252	2	165513091	T	C	0.59	0.068	0.005	6.00E-25
IRS1	rs2972144	2	227101411	G	A	0.64	0.095	0.005	2.10E-46
PPARG	rs11709077	3	12336507	G	A	0.88	0.131	0.009	1.80E-36
UBE2E2	rs35352848	3	23455582	T	C	0.79	0.068	0.009	1.30E-17
KIF9	rs11926707	3	46925539	C	T	0.63	0.239	0.042	2.10E-08
RBM6	rs4688760	3	49980596	T	C	0.68	0.039	0.010	3.50E-10
RFT1	rs2581787	3	53127677	T	G	0.56	0.039	0.005	2.40E-08
CACNA2D3	rs76263492	3	54828827	T	G	0.05	0.086	0.018	6.30E-09
PSMD6	rs3774723	3	63962339	G	A	0.84	0.068	0.009	1.60E-13
ADAMTS9	rs9860730	3	64701146	A	G	0.70	0.058	0.005	4.90E-15
SHQ1	rs13085136	3	72865183	C	T	0.93	0.077	0.009	1.50E-08
ROBO2	rs2272163	3	77671721	C	A	0.62	0.039	0.005	9.60E-09
ADCY5	rs11708067	3	123065778	A	G	0.77	0.086	0.009	5.20E-32
SLC12A8	rs649961	3	124926637	T	C	0.47	0.039	0.005	9.90E-10
TMCC1	rs9828772	3	129333182	C	G	0.90	0.058	0.010	4.20E-08
TSC22D2	rs62271373	3	150066540	A	T	0.06	0.086	0.014	1.00E-09
MBNL1	rs13065698	3	152086533	A	G	0.60	0.049	0.005	8.10E-13
EGFEM1P	rs7629630	3	168218841	A	T	0.86	0.049	0.010	2.50E-08
SLC2A2	rs9873618	3	170733076	G	A	0.71	0.068	0.005	4.80E-21
ABCC5	rs2872246	3	183738460	A	C	0.45	0.039	0.005	1.50E-08
IGF2BP2	rs6780171	3	185503456	A	T	0.31	0.131	0.009	9.00E-56
ST6GAL1	rs3887925	3	186665645	T	C	0.55	0.068	0.005	3.10E-22
LPP	rs4686471	3	187740899	C	T	0.61	0.058	0.010	1.70E-20
PCGF3	rs1531583	4	744972	T	G	0.05	0.122	0.013	3.50E-14
MAEA	rs56337234	4	1784403	C	T	0.50	0.058	0.005	8.60E-18
HTT	rs362307	4	3241845	T	C	0.08	0.077	0.009	1.10E-09
WFS1	rs10937721	4	6306763	C	G	0.59	0.058	0.010	1.50E-08
LCORL	rs12640250	4	17792869	C	A	0.71	0.039	0.005	3.70E-08

GNPDA2	rs10938398	4	45186139	A	G	0.43	0.049	0.005	3.60E-12
USP46	rs2102278	4	52818664	G	A	0.32	0.039	0.005	3.70E-08
SCD5	rs12642790	4	83578271	A	G	0.34	0.039	0.010	4.40E-10
FAM13A	rs1903002	4	89740894	G	C	0.50	0.039	0.005	2.70E-08
SMARCAD1	rs6821438	4	95091911	A	G	0.53	0.039	0.010	4.00E-11
SLC9B1	rs1580278	4	104140848	C	A	0.47	0.039	0.005	2.20E-10
PABPC4L	rs1296328	4	137083193	A	C	0.45	0.039	0.005	3.50E-08
TMEM154	rs7669833	4	153513369	T	A	0.70	0.058	0.005	1.20E-14
PDGFC	rs28819812	4	157652753	C	A	0.68	0.039	0.010	2.20E-08
ACSL1	rs58730668	4	185717759	T	C	0.86	0.068	0.009	1.30E-13
ANKH	rs146886108	5	14751305	C	T	0.99	0.344	0.048	7.80E-13
MRPS30	rs6884702	5	44682589	G	A	0.39	0.039	0.010	1.50E-10
ITGA1	rs3811978	5	52100489	G	A	0.17	0.058	0.005	7.70E-11
ARL15	rs702634	5	53271420	A	G	0.69	0.049	0.010	7.70E-14
ANKRD55	rs465002	5	55808475	T	C	0.74	0.104	0.005	6.10E-38
POC5	rs2307111	5	75003678	T	C	0.61	0.049	0.010	2.10E-16
ZBED3	rs4457053	5	76424949	G	A	0.30	0.058	0.010	8.40E-18
DMGDH	rs1316776	5	78430607	C	A	0.65	0.049	0.005	2.60E-12
RASA1	rs7719891	5	86577352	G	A	0.26	0.039	0.010	2.40E-08
SLCO6A1	rs138337556	5	101232944	G	A	0.00	0.445	0.076	4.70E-09
PAM	rs115505614	5	102422968	T	C	0.05	0.174	0.013	1.30E-30
PHF15	rs329122	5	133864599	A	G	0.43	0.039	0.005	3.60E-09
RREB1	rs9379084	6	7231843	G	A	0.89	0.104	0.009	3.30E-21
CDKAL1	rs7756992	6	20679709	G	A	0.27	0.140	0.009	2.40E-88
MHC	rs601945	6	32573415	G	A	0.18	0.058	0.010	4.70E-08
LRFN2	rs34298980	6	40409243	T	C	0.50	0.039	0.005	9.30E-10
VEGFA	rs6458354	6	43814190	C	T	0.29	0.049	0.010	2.10E-12
TFAP2B	rs3798519	6	50788778	C	A	0.18	0.058	0.010	2.60E-12
SLC25A51P1	rs555402748	6	67387490	T	C	0.00	1.300	0.239	4.60E-08
BEND3	rs4946812	6	107431688	G	A	0.67	0.039	0.005	8.20E-09
CENPW	rs11759026	6	126792095	G	A	0.23	0.068	0.005	2.40E-18
SOGA3	rs2800733	6	127416930	A	G	0.72	0.049	0.005	6.00E-11
SLC35D3	rs9494624	6	137300960	A	G	0.29	0.039	0.010	6.10E-09
SLC22A3	rs474513	6	160770312	A	G	0.52	0.039	0.005	8.10E-10
QKI	rs4709746	6	164133001	C	T	0.87	0.058	0.010	5.80E-09
DGKB	rs10228066	7	15063569	T	C	0.54	0.068	0.009	1.10E-28
IGF2BP3	rs4279506	7	23512896	G	C	0.61	0.058	0.010	4.80E-08
JAZF1	rs1708302	7	28198677	C	T	0.51	0.095	0.005	1.10E-48
CRHR2	rs917195	7	30728452	C	T	0.77	0.049	0.010	4.20E-11
GCK	rs878521	7	44255643	A	G	0.25	0.058	0.005	1.90E-13
FBXL13	rs11496066	7	102486254	T	C	0.82	0.077	0.014	1.10E-08
RELN	rs39328	7	103444978	T	C	0.43	0.039	0.005	3.70E-08
CTTNBP2	rs6976111	7	117495667	A	C	0.31	0.039	0.010	1.20E-08
KLF14	rs1562396	7	130457914	G	A	0.32	0.058	0.010	9.90E-18
AOC1	rs62492368	7	150537635	A	G	0.31	0.049	0.005	1.10E-10
MNX1	rs6459733	7	156930550	G	C	0.67	0.058	0.005	2.40E-17
MSRA	rs17689007	8	9974824	G	A	0.53	0.039	0.005	2.50E-09
XKR6	rs57327348	8	10808687	A	T	0.78	0.039	0.010	4.50E-08
LPL	rs10096633	8	19830921	C	T	0.88	0.068	0.009	1.10E-12
PURG	rs10954772	8	30863938	T	C	0.31	0.039	0.010	1.80E-09
ANK1	rs13262861	8	41508577	C	A	0.83	0.068	0.009	4.00E-12
TP53INP1	rs10097617	8	95961626	T	C	0.48	0.039	0.010	3.30E-11
CPQ	rs149364428	8	97737741	A	G	0.01	0.239	0.035	1.80E-12
TRHR	rs12680028	8	110123183	C	G	0.53	0.039	0.005	2.50E-08
SLC30A8	rs3802177	8	118185025	G	A	0.69	0.104	0.009	1.10E-55
CASC11	rs17772814	8	128711742	G	A	0.92	0.077	0.014	5.40E-10
PVT1	rs1561927	8	129568078	C	T	0.27	0.039	0.010	1.50E-09

BOP1	rs4977213	8	145507304	C	T	0.37	0.049	0.010	9.10E-14
GLIS3	rs10974438	9	4291928	C	A	0.36	0.049	0.010	1.50E-14
HAUS6	rs7022807	9	19067833	G	A	0.40	0.039	0.005	2.70E-10
FOCAD	rs7867635	9	20241069	C	T	0.41	0.039	0.005	4.00E-08
CDKN2A/B	rs10811660	9	22134068	G	A	0.83	0.239	0.008	1.40E-115
LINGO2	rs1412234	9	28410683	C	T	0.32	0.039	0.010	1.90E-10
UBAP2	rs12001437	9	34074476	C	T	0.37	0.039	0.010	2.80E-10
MTND2P8	rs11137820	9	81359113	C	G	0.58	0.039	0.005	2.90E-08
TLE4	rs17791513	9	81905590	A	G	0.93	0.095	0.014	3.10E-14
TLE1	rs2796441	9	84308948	G	A	0.59	0.068	0.005	4.40E-24
ZNF169	rs55653563	9	97001682	A	C	0.73	0.039	0.010	2.20E-09
ABO	rs505922	9	136149229	C	T	0.33	0.049	0.005	3.90E-12
GPSM1	rs28505901	9	139241030	G	A	0.75	0.086	0.009	6.70E-26
CDC123/CAMK1D	rs11257655	10	12307894	T	C	0.22	0.086	0.009	1.50E-32
NEUROG3	rs2642588	10	71466578	G	T	0.70	0.049	0.010	2.20E-14
ZMIZ1	rs703972	10	80952826	G	C	0.53	0.068	0.009	1.70E-29
HHEX/IDE	rs10882101	10	94462427	T	C	0.59	0.058	0.010	1.40E-08
TCF7L2	rs7903146	10	114758349	T	C	0.30	0.315	0.007	5.80E-447
PLEKHA1	rs2280141	10	124193181	T	G	0.52	0.049	0.005	1.40E-13
INS/IGF2	rs4929965	11	2197286	A	G	0.38	0.068	0.009	4.00E-26
KCNQ1	rs2237895	11	2857194	C	A	0.43	0.113	0.009	6.00E-52
PDE3B	rs141521721	11	14763828	A	C	0.02	0.122	0.018	2.70E-08
KCNJ11	rs5213	11	17408404	C	T	0.36	0.068	0.009	3.50E-27
QSER1	rs145678014	11	32927778	G	T	0.96	0.104	0.014	2.00E-10
PDHX	rs2767036	11	34982148	C	A	0.29	0.039	0.005	3.30E-08
HSD17B12	rs1061810	11	43877934	A	C	0.29	0.049	0.010	6.00E-13
CRY2	rs7115753	11	45912013	A	G	0.45	0.039	0.005	3.80E-09
CELF1	rs7124681	11	47529947	A	C	0.41	0.039	0.005	5.10E-09
MAP3K11	rs1783541	11	65294799	T	C	0.20	0.058	0.010	2.00E-14
CCND1	rs11820019	11	69448758	T	C	0.97	0.148	0.017	5.10E-12
CENTD2/ARAP1	rs77464186	11	72460398	A	C	0.84	0.104	0.009	4.70E-33
MTNR1B	rs10830963	11	92708710	G	C	0.28	0.095	0.009	4.80E-43
ETS1	rs67232546	11	128398938	T	C	0.21	0.058	0.005	1.30E-11
CCND2	rs76895963	12	4384844	T	G	0.98	0.482	0.028	1.40E-69
CDKN1B	rs2066827	12	12871099	G	T	0.24	0.049	0.005	4.20E-08
ITPR2	rs718314	12	26453283	G	A	0.25	0.049	0.005	8.40E-11
KLHDC5	rs10842994	12	27965150	C	T	0.81	0.077	0.005	4.10E-20
HMGA2	rs2258238	12	66221060	T	A	0.10	0.095	0.014	4.50E-21
TSPAN8/LGR5	rs1796330	12	71522953	G	C	0.57	0.049	0.005	2.20E-14
USP44	rs2197973	12	95928560	T	C	0.54	0.039	0.005	3.60E-08
RMST	rs77864822	12	97848775	A	G	0.93	0.077	0.014	1.10E-08
WSCD2	rs1426371	12	108629780	G	A	0.74	0.049	0.010	8.20E-12
KSR2	rs34965774	12	118412373	A	G	0.14	0.058	0.010	2.00E-09
HNF1A	rs56348580	12	121432117	G	C	0.69	0.049	0.010	2.30E-13
MPHOSPH9	rs4148856	12	123450765	C	G	0.78	0.049	0.010	1.70E-10
FBRSL1	rs12811407	12	133069698	A	G	0.33	0.049	0.010	1.70E-12
RNF6	rs34584161	13	26776999	A	G	0.76	0.049	0.005	2.20E-10
HMGB1	rs11842871	13	31042452	G	T	0.73	0.039	0.010	1.20E-08
KL	rs576674	13	33554302	G	A	0.17	0.049	0.010	8.30E-10
DLEU1	rs963740	13	51096095	A	T	0.71	0.039	0.005	2.10E-08
PCDH17	rs9537803	13	58366634	C	T	0.28	0.039	0.010	4.60E-08
SRGAP2D	rs9563615	13	59077406	A	T	0.71	0.049	0.005	6.40E-11
SPRY2	rs1359790	13	80717156	G	A	0.72	0.086	0.005	2.40E-31
IRS2	rs7987740	13	109947213	T	C	0.61	0.039	0.005	4.00E-08
SLC7A7	rs17122772	14	23288935	G	C	0.23	0.039	0.010	1.60E-08
AKAP6	rs17522122	14	33302882	T	G	0.47	0.039	0.005	3.20E-09
CLEC14A	rs8017808	14	38848419	G	T	0.74	0.039	0.010	2.10E-08

NRXN3	rs17836088	14	79932041	C	G	0.22	0.058	0.010	6.70E-14
SMEK1	rs8010382	14	91963722	G	A	0.42	0.039	0.005	6.50E-09
MARK3	rs62007683	14	103894071	G	T	0.65	0.039	0.005	3.10E-08
RASGRP1	rs34715063	15	38873115	C	T	0.12	0.095	0.009	2.30E-19
LTK	rs11070332	15	41809205	A	G	0.36	0.049	0.005	1.10E-13
ONECUT1	rs2456530	15	53091553	T	C	0.13	0.058	0.010	5.40E-09
WDR72	rs528350911	15	53747228	G	C	0.01	0.239	0.042	2.10E-08
TCF12	rs117483894	15	57456802	G	A	0.04	0.095	0.014	3.90E-08
C2CD4A/B	rs8037894	15	62394264	G	C	0.57	0.049	0.005	2.60E-13
USP3	rs7178762	15	63871292	C	T	0.46	0.039	0.005	5.40E-10
MAP2K5	rs4776970	15	68080886	A	T	0.64	0.039	0.005	5.00E-09
PTPN9	rs13737	15	75932129	G	T	0.76	0.049	0.005	5.60E-10
HMG20A	rs1005752	15	77818128	A	C	0.72	0.077	0.009	2.50E-29
AP3S2	rs4932265	15	90423293	T	C	0.27	0.068	0.005	4.20E-20
PRC1	rs12910825	15	91511260	G	A	0.36	0.049	0.010	1.60E-15
ITFG3	rs6600191	16	295795	T	C	0.82	0.058	0.010	9.30E-13
CLUAP1	rs3751837	16	3583173	T	C	0.22	0.039	0.010	1.40E-08
ATP2A1	rs8046545	16	28915217	G	A	0.36	0.039	0.005	1.90E-08
FAM57B	rs11642430	16	30045789	G	C	0.40	0.039	0.005	2.20E-09
FTO	rs1421085	16	53800954	C	T	0.42	0.122	0.009	3.10E-84
NFAT5	rs862320	16	69651866	C	T	0.58	0.039	0.010	3.90E-11
BCAR1	rs72802342	16	75234872	C	A	0.92	0.157	0.013	4.00E-32
CMIP	rs2925979	16	81534790	T	C	0.30	0.049	0.010	1.40E-14
SPG7	rs12920022	16	89564055	A	T	0.16	0.049	0.010	3.40E-09
ZZEF1	rs1377807	17	4045440	C	G	0.31	0.049	0.010	4.20E-13
GLP2R	rs7222481	17	9785187	C	G	0.32	0.039	0.005	1.40E-08
RAI1	rs4925109	17	17661802	A	G	0.32	0.049	0.005	2.80E-12
NF1	rs71372253	17	29413019	C	T	0.06	0.077	0.009	4.40E-08
HNF1B	rs10908278	17	36099952	T	A	0.48	0.077	0.009	6.40E-36
MLX	rs34855406	17	40731411	C	G	0.28	0.049	0.010	2.30E-12
TTLL6	rs35895680	17	47060322	C	A	0.68	0.058	0.005	2.50E-15
KIF2B	rs569511541	17	52140805	G	A	0.00	2.032	0.358	1.50E-08
ACE	rs60276348	17	62203304	T	C	0.14	0.049	0.010	2.60E-08
BPTF	rs61676547	17	65892507	C	G	0.19	0.058	0.005	2.90E-11
LAMA1	rs7240767	18	7070642	C	T	0.38	0.039	0.005	1.60E-08
COMMD9	rs62080313	18	36278709	C	T	0.12	0.058	0.010	1.00E-08
TCF4	rs72926932	18	53050646	C	A	0.08	0.086	0.014	1.00E-14
WDR7	rs17684074	18	54675384	G	C	0.74	0.039	0.010	2.90E-08
GRP	rs9957145	18	56876228	G	A	0.83	0.049	0.010	8.10E-09
MC4R	rs523288	18	57848369	T	A	0.24	0.049	0.010	7.60E-13
BCL2A	rs12454712	18	60845884	T	C	0.61	0.049	0.005	4.60E-13
UHRF1	rs7249758	19	4948862	A	G	0.20	0.049	0.010	3.40E-09
INSR	rs75253922	19	7240848	C	T	0.19	0.049	0.005	2.70E-08
MAP2K7	rs4804833	19	7970635	A	G	0.39	0.049	0.005	7.70E-13
FARSA	rs3111316	19	13038415	A	G	0.59	0.049	0.005	6.30E-13
TM6SF2	rs8107974	19	19388500	T	A	0.08	0.095	0.009	3.30E-15
PEPD	rs10406327	19	33890838	C	G	0.52	0.039	0.005	3.80E-08
TOMM40/APOE	rs429358	19	45411941	T	C	0.85	0.077	0.009	2.60E-18
GIPR	rs10406431	19	46157019	A	G	0.56	0.049	0.005	9.60E-14
ZC3H4	rs3810291	19	47569003	A	G	0.67	0.049	0.005	8.90E-12
NKX2.2	rs13041756	20	21466795	C	T	0.11	0.058	0.010	1.40E-08
RALY	rs2268078	20	32596704	A	G	0.66	0.039	0.010	2.30E-10
HNF4A	rs1800961	20	43042364	T	C	0.04	0.166	0.021	2.30E-22
EYA2	rs6063048	20	45598564	G	A	0.72	0.049	0.005	2.20E-11
CEBPB	rs11699802	20	48832135	C	T	0.54	0.039	0.010	1.80E-11
TSHZ2	rs34454109	20	51223594	A	T	0.77	0.039	0.010	7.10E-09
GNAS	rs6070625	20	57394628	G	C	0.52	0.049	0.005	5.30E-14

MTMR3/ASCC2	rs6518681	22	30609554	G	A	0.91	0.086	0.009	1.10E-12
YWHAH	rs117001013	22	32348841	C	T	0.91	0.068	0.009	1.70E-08
EP300	rs5758223	22	41489920	A	G	0.72	0.039	0.005	3.80E-08
PNPLA3	rs738408	22	44324730	T	C	0.23	0.049	0.010	1.40E-10
PIM3	rs1801645	22	50356850	C	T	0.28	0.039	0.005	1.50E-08

T2DM, type 2 diabetes mellitus; SNP, single nucleotide polymorphisms; CHR, chromosome; BP, physical position of SNP (base-pairs); A1, effect allele; A2, alternative allele; EAF: effect allele frequency; Beta, effect allele beta coefficient; SE, standard error.

ESM Table 2. Characteristics of genome-wide significant single nucleotide polymorphisms associated with T2DMadjBMI.

Nearest gene	SNP	CHR	BP	A1	A2	EAF	Beta	SE	*p*-value
MACF1	rs3768321	1	40035928	T	G	0.20	0.077	0.009	9.60E-16
FAF1	rs58432198	1	51256091	C	T	0.88	0.077	0.009	1.40E-09
PTGFRN	rs1127215	1	117532790	C	T	0.58	0.049	0.010	6.20E-11
NOTCH2	rs1493694	1	120526982	T	C	0.11	0.095	0.014	1.70E-15
PROX1	rs340874	1	214159256	C	T	0.56	0.068	0.009	4.80E-21
LYPLAL1	rs2820446	1	219748818	C	G	0.71	0.058	0.010	5.80E-14
ABCB10	rs348330	1	229672955	G	A	0.36	0.049	0.010	3.70E-11
GCKR	rs1260326	2	27730940	C	T	0.61	0.068	0.009	7.40E-19
THADA	rs6708643	2	43430440	A	G	0.50	0.049	0.010	3.40E-11
THADA	rs80147536	2	43698028	A	T	0.90	0.140	0.009	2.30E-26
BCL11A	rs243024	2	60583665	A	G	0.46	0.058	0.010	4.50E-15
CEP68	rs2028150	2	65655012	C	G	0.60	0.049	0.010	7.40E-11
GLI2	rs11688682	2	121347612	G	C	0.73	0.068	0.009	1.60E-13
CYTIP	rs13426680	2	158339550	A	G	0.94	0.095	0.014	1.40E-09
GRB14/COBLL1	rs10195252	2	165513091	T	C	0.59	0.077	0.009	5.70E-26
CRYBA2	rs113414093	2	219859171	A	G	0.05	0.113	0.022	6.60E-09
IRS1	rs2972144	2	227101411	G	A	0.64	0.104	0.009	2.90E-40
PPARG	rs11709077	3	12336507	G	A	0.88	0.122	0.009	5.00E-25
UBE2E2	rs35352848	3	23455582	T	C	0.79	0.086	0.009	5.40E-22
KIF9	rs11926707	3	46925539	C	T	0.63	0.049	0.005	1.70E-09
CACNA2D3	rs76263492	3	54828827	T	G	0.05	0.104	0.018	1.40E-08
PSMD6	rs3774723	3	63962339	G	A	0.84	0.068	0.009	1.70E-10
ADAMTS9	rs9860730	3	64701146	A	G	0.70	0.068	0.009	4.10E-16
SHQ1	rs13085136	3	72865183	C	T	0.93	0.086	0.014	5.60E-09
ADCY5	rs11708067	3	123065778	A	G	0.77	0.104	0.009	3.10E-33
TSC22D2	rs62271373	3	150066540	A	T	0.06	0.095	0.018	2.50E-09
MBNL1	rs74653713	3	152417881	C	A	0.96	0.131	0.018	8.30E-11
SLC2A2	rs9873618	3	170733076	G	A	0.71	0.077	0.009	2.70E-21
IGF2BP2	rs6780171	3	185503456	A	T	0.31	0.122	0.009	2.40E-51
LPP	rs4686471	3	187740899	C	T	0.61	0.068	0.009	1.10E-19
PCGF3	rs1531583	4	744972	T	G	0.05	0.131	0.018	8.80E-12
MAEA	rs56337234	4	1784403	C	T	0.50	0.077	0.005	1.30E-21
WFS1	rs1801212	4	6302519	A	G	0.71	0.095	0.009	1.30E-31
TMEM154	rs7669833	4	153513369	T	A	0.70	0.058	0.010	4.40E-13
ACSL1	rs58730668	4	185717759	T	C	0.86	0.068	0.014	2.50E-11
ANKH	rs17250977	5	14753745	G	A	0.04	0.148	0.022	9.50E-14
ANKH	rs6885132	5	14768092	C	G	0.90	0.095	0.014	6.60E-14
ARL15	rs702634	5	53271420	A	G	0.69	0.058	0.010	9.70E-14
ANKRD55	rs465002	5	55808475	T	C	0.74	0.077	0.009	7.60E-21
PIK3R1	rs4976033	5	67714246	G	A	0.41	0.049	0.005	1.00E-09
ZBED3	rs4457053	5	76424949	G	A	0.30	0.068	0.009	1.50E-17
PAM	rs115505614	5	102422968	T	C	0.05	0.191	0.017	3.80E-27
PHF15	rs329122	5	133864599	A	G	0.43	0.058	0.005	2.00E-13
EBF1	rs3934712	5	157928196	C	T	0.21	0.049	0.010	3.20E-08
RREB1	rs112498319	6	7035734	C	A	0.41	0.049	0.005	2.30E-09
RREB1	rs9379084	6	7231843	G	A	0.89	0.122	0.013	3.80E-23
CDKAL1	rs7756992	6	20679709	G	A	0.27	0.148	0.009	1.40E-73
MHC	rs601945	6	32573415	G	A	0.18	0.086	0.009	1.50E-17
HMGA1	rs77136196	6	34247047	T	C	0.04	0.113	0.018	1.60E-09
VEGFA	rs11967262	6	43760327	G	C	0.49	0.058	0.005	3.70E-13
VEGFA	rs6458354	6	43814190	C	T	0.29	0.049	0.010	7.40E-11
CENPW	rs11759026	6	126792095	G	A	0.23	0.077	0.009	1.20E-16
SOGA3	rs2800733	6	127416930	A	G	0.72	0.068	0.005	2.40E-14
MIR3668	rs2982521	6	139835329	A	T	0.38	0.049	0.005	8.80E-09

SLC22A3	rs474513	6	160770312	A	G	0.52	0.049	0.010	1.30E-11
QKI	rs4709746	6	164133001	C	T	0.87	0.077	0.009	3.50E-11
DGKB	rs17168486	7	14898282	T	C	0.18	0.077	0.009	7.50E-14
DGKB	rs10228066	7	15063569	T	C	0.54	0.068	0.009	2.20E-21
IGF2BP3	rs4279506	7	23512896	G	C	0.61	0.039	0.010	2.80E-08
JAZF1	rs1708302	7	28198677	C	T	0.51	0.104	0.005	8.70E-41
GCK	rs878521	7	44255643	A	G	0.25	0.058	0.010	8.70E-13
KLF14	rs1562396	7	130457914	G	A	0.32	0.068	0.009	1.10E-16
AOC1	rs62492368	7	150537635	A	G	0.31	0.049	0.010	3.80E-10
MNX1	rs6459733	7	156930550	G	C	0.67	0.058	0.010	1.00E-14
LPL	rs10096633	8	19830921	C	T	0.88	0.077	0.009	2.10E-10
ANK1	rs13262861	8	41508577	C	A	0.83	0.104	0.014	7.40E-27
ANK1	rs148766658	8	41552046	C	T	0.04	0.131	0.022	1.40E-10
TP53INP1	rs11786992	8	95685147	A	C	0.64	0.049	0.010	1.10E-11
SLC30A8	rs3802177	8	118185025	G	A	0.69	0.122	0.009	3.80E-53
CASC11	rs17772814	8	128711742	G	A	0.92	0.086	0.014	4.00E-08
BOP1	rs4977213	8	145507304	C	T	0.37	0.058	0.010	1.20E-15
GLIS3	rs10974438	9	4291928	C	A	0.36	0.058	0.010	3.20E-14
HAUS6	rs7022807	9	19067833	G	A	0.40	0.049	0.005	3.20E-10
CDKN2A/B	rs76011118	9	22133773	A	G	0.03	0.223	0.024	2.70E-22
CDKN2A/B	rs10811660	9	22134068	G	A	0.83	0.174	0.013	5.90E-69
TLE4	rs17791513	9	81905590	A	G	0.93	0.104	0.014	8.10E-12
TLE1	rs2796441	9	84308948	G	A	0.59	0.077	0.009	1.30E-25
GPSM1	rs28505901	9	139241030	G	A	0.75	0.077	0.009	2.80E-19
CDC123/CAMK1D	rs11257655	10	12307894	T	C	0.22	0.095	0.009	7.70E-26
NEUROG3	rs41277236	10	71332301	T	C	0.04	0.122	0.018	1.70E-09
NEUROG3	rs2642588	10	71466578	G	T	0.70	0.049	0.010	1.20E-09
ZMIZ1	rs703972	10	80952826	G	C	0.53	0.077	0.009	3.40E-26
PTEN	rs11202627	10	89769340	T	C	0.15	0.058	0.010	4.70E-08
HHEX/IDE	rs10882101	10	94462427	T	C	0.59	0.122	0.009	1.60E-57
TCF7L2	rs184509201	10	114740337	C	G	0.98	0.199	0.028	1.10E-11
TCF7L2	rs7903146	10	114758349	T	C	0.30	0.358	0.011	0.00E+00
WDR11	rs72631105	10	122915345	A	G	0.19	0.058	0.010	3.70E-09
PLEKHA1	rs2280141	10	124193181	T	G	0.52	0.049	0.005	3.70E-10
INS/IGF2	rs4929965	11	2197286	A	G	0.38	0.068	0.009	1.60E-19
KCNQ1	rs231349	11	2672821	T	C	0.10	0.068	0.014	4.00E-08
KCNQ1	rs231361	11	2691500	A	G	0.26	0.077	0.009	6.90E-18
KCNQ1	rs2237895	11	2857194	C	A	0.43	0.113	0.005	9.90E-47
METTL15	rs4923543	11	28534898	A	G	0.33	0.039	0.010	4.50E-08
MAP3K11	rs1783541	11	65294799	T	C	0.20	0.058	0.005	4.40E-09
CCND1	rs11820019	11	69448758	T	C	0.97	0.140	0.026	1.40E-08
CENTD2/ARAP1	rs77464186	11	72460398	A	C	0.84	0.131	0.009	9.00E-37
MTNR1B	rs10830963	11	92708710	G	C	0.28	0.104	0.009	2.80E-36
MTNR1B	rs57235767	11	93013531	C	T	0.71	0.049	0.010	9.30E-09
ETS1	rs10893829	11	128042575	T	C	0.85	0.058	0.010	7.00E-09
ETS1	rs10750397	11	128234144	A	G	0.28	0.049	0.010	9.80E-09
ETS1	rs67232546	11	128398938	T	C	0.21	0.058	0.010	9.50E-10
CCND2	rs3217792	12	4384696	C	T	0.91	0.131	0.018	7.50E-22
CCND2	rs76895963	12	4384844	T	G	0.98	0.525	0.032	1.30E-64
CDKN1B	rs2066827	12	12871099	G	T	0.24	0.049	0.010	4.40E-08
ITPR2	rs718314	12	26453283	G	A	0.25	0.058	0.010	3.00E-12
KLHDC5	rs10842994	12	27965150	C	T	0.81	0.077	0.009	2.00E-16
HMGA2	rs2258238	12	66221060	T	A	0.10	0.113	0.013	1.90E-21
TSPAN8/LGR5	rs1796330	12	71522953	G	C	0.57	0.049	0.010	1.00E-11
WSCD2	rs1426371	12	108629780	G	A	0.74	0.049	0.010	3.50E-09
HNF1A	rs73226260	12	121380541	G	A	0.97	0.122	0.022	4.30E-09
HNF1A	rs1800574	12	121416864	T	C	0.03	0.148	0.022	5.70E-12

MPHOSPH9	rs4148856	12	123450765	C	G	0.78	0.058	0.005	2.40E-09
ZNF664	rs7978610	12	124468572	G	C	0.67	0.049	0.010	7.70E-10
ZNF664	rs825452	12	124509177	A	G	0.60	0.039	0.010	8.90E-09
RNF6	rs34584161	13	26776999	A	G	0.76	0.049	0.010	5.20E-09
DLEU1	rs963740	13	51096095	A	T	0.71	0.049	0.005	1.40E-08
SPRY2	rs1359790	13	80717156	G	A	0.72	0.095	0.005	6.50E-28
SLC7A7	rs17122772	14	23288935	G	C	0.23	0.049	0.010	8.00E-09
CLEC14A	rs8017808	14	38848419	G	T	0.74	0.049	0.010	3.70E-08
RASGRP1	rs34715063	15	38873115	C	T	0.12	0.086	0.009	3.70E-13
LTK	rs11070332	15	41809205	A	G	0.36	0.049	0.010	9.60E-10
C2CD4A/B	rs8037894	15	62394264	G	C	0.57	0.049	0.005	4.40E-10
USP3	rs7178762	15	63871292	C	T	0.46	0.039	0.010	4.40E-09
PTPN9	rs13737	15	75932129	G	T	0.76	0.049	0.010	1.10E-08
HMG20A	rs1005752	15	77818128	A	C	0.72	0.068	0.009	7.80E-18
AP3S2	rs4932265	15	90423293	T	C	0.27	0.077	0.009	7.70E-20
ITFG3	rs6600191	16	295795	T	C	0.82	0.068	0.009	3.80E-11
FTO	rs1421085	16	53800954	C	T	0.42	0.058	0.005	9.90E-13
BCAR1	rs72802342	16	75234872	C	A	0.92	0.157	0.017	2.70E-29
CMIP	rs2925979	16	81534790	T	C	0.30	0.049	0.010	2.50E-09
ZZEF1	rs1377807	17	4045440	C	G	0.31	0.049	0.010	2.70E-11
ATP1B2	rs1641523	17	7549681	C	T	0.43	0.039	0.010	8.30E-09
RAI1	rs4925109	17	17661802	A	G	0.32	0.049	0.005	5.30E-09
HNF1B	rs10908278	17	36099952	T	A	0.48	0.086	0.009	2.60E-31
BCL2A	rs12454712	18	60845884	T	C	0.61	0.068	0.009	5.60E-19
INSR	rs75253922	19	7240848	C	T	0.19	0.058	0.010	1.10E-08
MAP2K7	rs4804833	19	7970635	A	G	0.39	0.058	0.005	1.60E-12
FARSA	rs3111316	19	13038415	A	G	0.59	0.049	0.010	1.60E-11
TM6SF2	rs8107974	19	19388500	T	A	0.08	0.122	0.013	8.00E-18
PEPD	rs10406327	19	33890838	C	G	0.52	0.049	0.005	3.00E-09
GIPR	rs2238689	19	46178661	C	T	0.42	0.077	0.005	1.40E-21
HNF4A	rs4810426	20	43001721	T	C	0.11	0.095	0.014	2.40E-16
HNF4A	rs1800961	20	43042364	T	C	0.04	0.182	0.021	9.20E-18
EYA2	rs6063048	20	45598564	G	A	0.72	0.049	0.010	2.40E-10
GNAS	rs6070625	20	57394628	G	C	0.52	0.049	0.010	1.40E-11
TCEA2	rs59944054	20	62693175	A	G	0.24	0.049	0.010	4.60E-08
MTMR3/ASCC2	rs6518681	22	30609554	G	A	0.91	0.086	0.014	2.00E-10
YWHAH	rs117001013	22	32348841	C	T	0.91	0.077	0.014	8.00E-09
PNPLA3	rs738408	22	44324730	T	C	0.23	0.058	0.010	3.00E-12
PIM3	rs1801645	22	50356850	C	T	0.28	0.058	0.010	1.90E-10

T2DMadjBMI: type 2 diabetes mellitus adjusted for BMI; SNP, single nucleotide polymorphisms; CHR, chromosome; BP, physical position of SNP (base-pairs); A1, effect allele; A2, alternative allele; EAF: effect allele frequency; Beta, effect allele beta coefficient; SE, standard error.

ESM Table 3. Characteristics of genome-wide significant single-nucleotide polymorphisms associated with $FG_{adj}BMI$.

Nearest Gene	SNP	CHR	BP	A1	A2	EAF	Beta	SE	*p*-value
CDC14A	rs6662924	1	100894419	A	C	0.19	0.014	0.002	3.34E-10
ARNT	rs78132593	1	150868102	A	C	0.21	-0.015	0.002	2.60E-10
PROX1-AS1	rs2075423	1	214154719	T	G	0.37	-0.016	0.002	3.18E-21
ABCB10	rs348330	1	229672955	A	G	0.63	-0.012	0.002	3.04E-10
DPYSL5	rs877273	2	27140022	T	C	0.38	0.013	0.002	7.22E-15
GCKR	rs1260326	2	27730940	T	C	0.39	-0.028	0.002	4.48E-65
THADA	rs183381538	2	43775309	A	C	0.07	-0.025	0.004	7.81E-15
EML6	rs189548	2	54941112	A	G	0.73	-0.012	0.002	2.81E-09
G6PC2	rs111485380	2	169754123	T	C	0.83	0.070	0.003	1.02E-137
G6PC2	rs492594	2	169764176	C	G	0.46	0.016	0.002	7.36E-24
G6PC2	rs145353824	2	169765277	A	C	0.98	-0.076	0.009	7.21E-18
G6PC2	rs17539351	2	169766560	T	C	0.12	-0.068	0.003	5.86E-123
G6PC2	rs13430620	2	169768891	A	C	0.96	0.073	0.006	3.43E-40
ABCB11	rs114764002	2	169776141	A	T	0.97	0.092	0.007	3.41E-43
ABCB11	rs56100844	2	169786707	T	G	0.98	0.118	0.010	9.82E-35
ABCB11	rs3755158	2	169792188	C	G	0.89	-0.040	0.003	4.83E-49
ABCB11	rs114691375	2	169813318	A	T	0.03	0.045	0.006	7.79E-17
ADCY5	rs11708067	3	123065778	A	G	0.77	0.028	0.002	1.63E-43
ZBTB38	rs16851397	3	141134818	A	G	0.95	0.033	0.004	1.26E-12
MBNL1	rs17437560	3	152180329	T	C	0.11	-0.018	0.003	3.33E-08
SLC2A2	rs1604038	3	170709193	T	C	0.29	-0.020	0.002	4.47E-28
LPP-AS2	rs6808574	3	187740523	T	C	0.39	-0.013	0.002	7.21E-14
ACSL1	rs4862423	4	185726548	T	C	0.40	0.012	0.002	4.45E-10
LOC101928448	rs157512	5	55809127	T	C	0.76	0.013	0.002	5.43E-10
ZBED3-AS1	rs7708285	5	76425867	A	G	0.73	-0.013	0.002	1.25E-09
PCSK1	rs1820176	5	95696585	T	C	0.71	0.025	0.002	1.91E-34
LINC00491	rs7729395	5	102100576	T	C	0.05	-0.023	0.005	3.09E-08
RREB1	rs3778321	6	7250270	A	G	0.19	-0.019	0.002	3.16E-17
CDKAL1	rs9348441	6	20680678	A	T	0.28	0.018	0.002	4.40E-20
GLP1R	rs10305457	6	39034095	T	C	0.09	0.024	0.003	1.21E-14
GLP1R	rs10305492	6	39046794	A	G	0.01	-0.076	0.010	7.50E-16
RGS17	rs12055786	6	153431125	T	C	0.42	0.012	0.002	1.17E-11
DGKB	rs10281892	7	14919852	A	G	0.82	-0.028	0.002	2.09E-35
AGMO	rs10487796	7	15063430	A	T	0.48	-0.026	0.002	4.62E-52
GCK	rs2971671	7	44211337	T	C	0.77	-0.043	0.002	1.24E-104
GCK	rs3757840	7	44231216	T	G	0.49	0.042	0.002	5.94E-133
GCK	rs6975024	7	44231886	T	C	0.84	-0.062	0.002	4.66E-167
YKT6	rs138917529	7	44235694	A	T	0.98	0.060	0.007	4.08E-17
CAMK2B	rs878521	7	44255643	A	G	0.24	0.055	0.002	2.65E-174
GRB10	rs2108349	7	50786663	A	G	0.66	-0.016	0.002	1.25E-15
STYXL1	rs58925536	7	75654574	T	C	0.03	0.031	0.005	5.82E-09
STEAP2-AS1	rs13242882	7	89800053	A	T	0.46	-0.010	0.002	9.27E-09
LOC157273	rs7012637	8	9173209	A	G	0.47	-0.018	0.002	9.75E-25
LOC157273	rs9987289	8	9183358	A	G	0.10	0.028	0.003	3.57E-23
TPD52	rs12541643	8	81076874	T	C	0.48	0.012	0.002	4.51E-09
TP53INP1	rs896854	8	95960511	T	C	0.50	0.010	0.002	5.61E-09
SLC30A8	rs9650069	8	118204020	T	C	0.32	-0.029	0.002	8.31E-58
GLIS3	rs10974438	9	4291928	A	C	0.64	-0.020	0.002	9.85E-31
CDKN2B-AS1	rs10811660	9	22134068	A	G	0.18	-0.022	0.002	7.94E-25
IKBKAP	rs16913693	9	111680359	T	G	0.97	0.039	0.005	2.82E-16
ABO	rs507666	9	136149399	A	G	0.20	0.016	0.002	6.99E-17
DNLZ	rs3829109	9	139256766	A	G	0.30	-0.016	0.002	1.09E-15
GAD2	rs2839671	10	26505822	A	G	0.17	-0.016	0.002	8.38E-14
PDE6C	rs7095788	10	95384152	T	C	0.35	-0.011	0.002	1.98E-09

ADRA2A	rs12784552	10	113036354	A	G	0.91	0.033	0.003	2.86E-31
TCF7L2	rs7903146	10	114758349	T	C	0.27	0.026	0.002	2.00E-35
INS; INS-IGF2	rs3842753	11	2181060	T	G	0.28	0.013	0.002	2.84E-09
KCNQ1	rs4930011	11	2856658	C	G	0.59	-0.009	0.002	2.84E-08
LMO1	rs2168101	11	8255408	A	C	0.32	-0.013	0.002	3.09E-08
CRY2	rs10838524	11	45870177	A	G	0.45	0.024	0.002	1.56E-40
MADD	rs10501320	11	47293799	C	G	0.25	-0.022	0.002	1.09E-29
FADS2	rs174583	11	61609750	T	C	0.36	-0.017	0.002	3.37E-22
ARAP1	rs11603349	11	72460694	T	C	0.82	0.024	0.002	3.12E-25
MTNR1B	rs7113297	11	92671744	T	C	0.21	0.057	0.003	7.09E-100
MTNR1B	rs11523890	11	92679778	T	C	0.34	0.052	0.002	6.75E-161
MTNR1B	rs10466351	11	92697981	T	C	0.38	0.056	0.002	1.14E-205
MTNR1B	rs10830962	11	92698427	C	G	0.60	-0.048	0.002	6.62E-156
MTNR1B	rs79354397	11	92708961	T	C	0.97	-0.081	0.006	4.82E-45
GLS2	rs2657879	12	56865338	A	G	0.82	-0.012	0.002	7.33E-09
RMST	rs6538804	12	97848910	C	G	0.61	0.014	0.002	9.41E-14
KDM2B	rs6489811	12	121893626	A	G	0.47	-0.011	0.002	3.27E-09
FBRSL1	rs11610045	12	133063768	A	G	0.50	0.014	0.002	3.26E-13
PDX1-AS1	rs11619319	13	28487599	A	G	0.78	-0.017	0.002	3.41E-20
KL	rs576674	13	33554302	A	G	0.84	-0.018	0.002	9.70E-13
FOXN3	rs35889227	14	90055468	T	G	0.62	-0.013	0.002	3.37E-10
WARS	rs12888855	14	100830818	A	C	0.21	-0.014	0.002	6.02E-12
C2CD4A	rs7163757	15	62391608	T	C	0.45	-0.022	0.002	2.64E-36
LMAN1L	rs12594062	15	75102851	T	C	0.36	0.010	0.002	4.64E-09
HMG20A	rs7178572	15	77747190	A	G	0.30	-0.012	0.002	7.09E-10
IGF1R	rs6598541	15	99271135	A	G	0.36	0.011	0.002	4.12E-12
ADCY9	rs2238435	16	4014282	C	G	0.40	0.011	0.002	3.82E-09
QPCTL	rs2302593	19	46196634	C	G	0.50	0.011	0.002	5.67E-10
LINC00261	rs6113722	20	22557099	A	G	0.04	-0.042	0.004	7.66E-25
ZHX3	rs17265513	20	39832628	T	C	0.79	-0.016	0.002	5.10E-14
MTMR3	rs39713	22	30343186	T	C	0.09	-0.017	0.003	1.77E-08

SNP, single nucleotide polymorphisms; CHR, chromosome; BP, physical position of SNP (base-pairs); A1, effect allele; A2, alternative allele; EAF: effect allele frequency; Beta, effect allele beta coefficient; SE, standard error.

ESM Table 4. Characteristics of genome-wide significant single nucleotide polymorphisms associated with $FI_{adj}BMI$.

Nearest Gene	SNP	CHR	BP	A1	A2	EAF	Beta	SE	*p*-value
LYPLAL1	rs6674544	1	219628973	A	G	0.57	0.018	0.002	6.97E-21
TMEM18	rs77935490	2	630902	A	T	0.21	0.014	0.003	1.07E-08
GCKR	rs1260326	2	27730940	T	C	0.39	-0.023	0.002	8.42E-38
COBLL1	rs75265117	2	165518799	C	G	0.88	0.028	0.003	1.54E-24
COBLL1	rs13389219	2	165528876	T	C	0.39	-0.020	0.002	5.84E-28
LOC646736	rs2943646	2	227099534	A	G	0.37	-0.025	0.002	8.47E-39
SYN2	rs308971	3	12116620	A	G	0.87	-0.022	0.003	3.91E-13
PPARG	rs35000407	3	12351521	T	G	0.86	0.026	0.003	1.50E-21
ITIH3	rs17331151	3	52844534	T	C	0.11	-0.016	0.003	1.52E-08
ADCY5	rs11708067	3	123065778	A	G	0.78	-0.014	0.002	1.30E-09
LINC01214	rs62271373	3	150066540	A	T	0.06	0.026	0.005	1.60E-08
FAM13A	rs3775380	4	89739808	A	G	0.50	-0.012	0.002	1.48E-11
TET2	rs9884482	4	106081636	T	C	0.61	-0.013	0.002	2.88E-11
HHIP	rs11727676	4	145659064	T	C	0.91	-0.020	0.004	2.90E-08
PDGFC	rs6855363	4	157670537	T	C	0.68	0.013	0.002	4.04E-08
ARL15	rs4865796	5	53272664	A	G	0.68	0.017	0.002	7.33E-17
LOC101928448	rs459193	5	55806751	A	G	0.27	-0.018	0.002	1.12E-18
LOC101928448	rs3936511	5	55860781	A	G	0.82	-0.019	0.003	2.81E-14
C6orf1	rs116141873	6	34222201	T	G	0.04	0.043	0.006	1.42E-11
RPS10-NUDT3; NUDT3	rs2780215	6	34236973	A	G	0.96	0.039	0.006	1.06E-09
VEGFA	rs998584	6	43757896	A	C	0.49	0.012	0.002	2.31E-10
LINC01512	rs9472135	6	43809802	T	C	0.70	0.011	0.002	4.21E-08
RSPO3	rs1474696	6	127449246	A	G	0.49	-0.015	0.002	3.02E-16
QKI	rs73013411	6	164126233	A	C	0.13	-0.018	0.003	2.08E-08
GRB10	rs2108349	7	50786663	A	G	0.66	-0.012	0.002	1.13E-08
KLF14	rs972283	7	130466854	A	G	0.47	-0.011	0.002	1.09E-08
PPP1R3B	rs330945	8	9021933	T	C	0.63	0.014	0.002	1.82E-11
LOC157273	rs7012814	8	9173358	A	G	0.48	-0.022	0.002	8.34E-30
LOC157273	rs4841132	8	9183596	A	G	0.11	0.026	0.003	3.83E-20
NKX2-6	rs13258890	8	23615445	T	C	0.77	0.013	0.003	2.77E-08
ABO	rs75179845	9	136132954	T	C	0.91	-0.022	0.004	6.05E-11
PTEN	rs118164457	10	89680631	T	C	0.96	-0.035	0.006	3.86E-10
TCF7L2	rs7903146	10	114758349	T	C	0.27	-0.012	0.002	1.24E-09
MACROD1	rs2845885	11	63869062	T	C	0.93	-0.020	0.004	1.18E-08
GYS2	rs6487237	12	21699928	A	C	0.78	0.015	0.003	4.68E-09
HDAC7	rs111264094	12	48202696	C	G	0.97	0.057	0.009	1.64E-09
HMGA2	rs1351394	12	66351826	T	C	0.49	-0.011	0.002	2.71E-09
IGF1	rs860598	12	102898446	A	G	0.82	0.018	0.003	6.88E-12
DNAH10	rs7133378	12	124409502	A	G	0.32	-0.013	0.002	6.00E-11
BCL2	rs12454712	18	60845884	T	C	0.58	0.014	0.003	1.78E-09
PEPD	rs731839	19	33899065	A	G	0.66	-0.012	0.002	3.87E-11
EYA2	rs1206760	20	45582472	A	G	0.54	-0.011	0.002	8.82E-10

$FG_{adj}BMI$, fasting glucose adjusted for BMI; SNP, single nucleotide polymorphisms; CHR, chromosome; BP, physical position of SNP (base-pairs); A1, effect allele; A2, alternative allele; EAF: effect allele frequency; Beta, effect allele beta coefficient; SE, standard error.

ESM Table 5. Characteristics of genome-wide significant single-nucleotide polymorphisms associated with HbA1c.

Nearest Gene	SNP	CHR	BP	A1	A2	EAF	Beta	SE	*p*-value
SMIM1	rs1175549	1	3691727	A	C	0.75	0.010	0.002	7.13E-13
SYF2	rs2375278	1	25529038	A	G	0.17	0.011	0.002	1.05E-11
CERS2	rs267738	1	150940625	T	G	0.79	0.011	0.002	1.14E-11
FDPS	rs7534795	1	155275553	T	C	0.26	0.010	0.002	2.13E-09
SPTA1	rs857725	1	158607935	T	G	0.73	-0.021	0.001	5.43E-55
ATP2B4	rs7547793	1	203653544	A	C	0.13	-0.012	0.002	6.61E-09
PROX1-AS1	rs340882	1	214145731	C	G	0.41	-0.008	0.001	1.48E-10
ATAD2B	rs12612492	2	24093756	T	C	0.12	0.019	0.002	1.88E-26
MFSD2B	rs6545222	2	24235704	A	G	0.75	0.010	0.002	1.86E-14
ZFP36L2	rs1367173	2	43449385	T	C	0.12	-0.015	0.002	1.66E-14
FOXN2	rs17037289	2	48587198	A	G	0.75	-0.009	0.002	2.40E-09
G6PC2	rs560887	2	169763148	T	C	0.30	-0.031	0.001	5.55E-122
ABCB11	rs56100844	2	169786707	T	G	0.98	0.058	0.007	4.47E-17
SCRN3	rs17256082	2	175292364	T	C	0.67	-0.007	0.001	3.19E-08
PNKD; TMBIM1	rs4674280	2	219141458	C	G	0.57	0.008	0.001	5.48E-09
SYN2	rs12491937	3	12268244	A	G	0.58	0.009	0.001	1.42E-13
USP4	rs9818758	3	49382925	A	G	0.18	0.013	0.002	1.49E-13
ADCY5	rs11719201	3	123068744	T	C	0.24	-0.013	0.002	2.43E-18
SLC2A2	rs1604038	3	170709193	T	C	0.29	-0.011	0.001	2.76E-16
PLD1	rs4894769	3	171516306	A	T	0.43	-0.007	0.001	3.61E-09
FNDC3B	rs13089972	3	171798694	A	T	0.58	0.011	0.001	1.87E-15
FREM3	rs13134327	4	144659795	A	G	0.33	0.014	0.001	2.81E-26
GYPA	rs112578089	4	145128105	A	G	0.04	-0.036	0.006	2.18E-11
MIR1303	rs6877043	5	154048367	T	C	0.64	0.009	0.001	1.99E-10
HAVCR1	rs1948759	5	156442657	A	G	0.17	-0.010	0.002	2.44E-08
RREB1	rs3778321	6	7250270	A	G	0.19	-0.011	0.002	4.18E-11
CDKAL1	rs6931514	6	20703952	A	G	0.73	-0.010	0.001	1.18E-13
HFE	rs1799945	6	26091179	C	G	0.86	0.025	0.002	3.32E-47
HFE	rs1800562	6	26093141	A	G	0.06	-0.038	0.003	2.33E-50
NFKBIL1	rs6929796	6	31522669	A	G	0.17	-0.009	0.002	3.26E-08
HBS1L	rs9376090	6	135411228	T	C	0.73	0.025	0.001	1.90E-62
DGKB	rs10231021	7	15060429	A	T	0.51	0.009	0.001	8.69E-14
GCK	rs10259649	7	44219705	T	C	0.77	-0.024	0.002	4.02E-55
GCK	rs2971670	7	44226101	T	C	0.17	0.032	0.002	5.10E-88
GCK	rs3757840	7	44231216	T	G	0.50	0.022	0.001	4.25E-71
MLXIPL	rs13234131	7	73025975	A	G	0.87	-0.011	0.002	2.06E-09
ASB15	rs4731113	7	123283949	T	C	0.96	0.020	0.004	4.90E-08
ANK1	rs34664882	8	41543675	A	G	0.03	-0.049	0.004	4.82E-37
SLC20A2	rs6980507	8	42383084	A	G	0.40	0.011	0.001	8.15E-20
SLC30A8	rs11558471	8	118185733	A	G	0.68	0.015	0.001	3.38E-25
TRIB1	rs2954021	8	126482077	A	G	0.49	-0.007	0.001	1.92E-10
CDKN2B-AS1	rs10811661	9	22134094	T	C	0.82	0.013	0.002	1.74E-14
VPS13A	rs12351997	9	80015424	T	C	0.80	-0.013	0.002	4.50E-14
C9orf47	rs61750929	9	91495135	T	C	0.06	-0.028	0.003	9.49E-24
KLF4	rs7042939	9	110511408	A	G	0.40	0.010	0.001	1.50E-15
ABO	rs649129	9	136154304	T	C	0.22	0.011	0.002	3.28E-15
DNLZ	rs3829109	9	139256766	A	G	0.30	-0.009	0.002	2.68E-08
CDC123	rs11257655	10	12307894	T	C	0.22	0.011	0.002	1.91E-13
HKDC1	rs5785903	10	71002040	T	TG	0.43	0.017	0.002	4.39E-29
HK1	rs4745982	10	71089843	T	G	0.92	0.074	0.003	8.36E-143
HK1	rs150705486	10	71093216	A	G	0.02	-0.114	0.006	4.24E-72
TCF7L2	rs7903146	10	114758349	T	C	0.28	0.013	0.001	1.04E-22
SIRT3	rs4980325	11	234451	T	G	0.53	0.011	0.001	4.70E-14
INS; INS-IGF2	rs3842753	11	2181060	T	G	0.28	0.008	0.002	3.93E-08

LOC440028	rs360140	11	9776567	A	C	0.66	-0.008	0.001	9.62E-13
MYBPC3	rs10838696	11	47363285	A	G	0.35	-0.007	0.001	6.53E-09
FADS1	rs174559	11	61581656	A	G	0.27	-0.011	0.001	3.31E-13
MTNR1B	rs10830963	11	92708710	C	G	0.72	-0.020	0.002	1.54E-36
ARHGAP42	rs11224302	11	100456604	T	C	0.10	-0.016	0.002	4.63E-14
CCND2-AS1	rs117233107	12	4328521	A	G	0.02	-0.047	0.007	8.45E-11
PHB2	rs2110073	12	7075882	T	C	0.10	0.012	0.002	5.79E-09
SENP1	rs76261711	12	48486696	A	T	0.90	0.014	0.002	4.35E-10
PFKM	rs4760682	12	48512285	A	C	0.80	0.016	0.002	3.20E-20
SH2B3	rs10774624	12	111833788	A	G	0.52	0.009	0.001	4.17E-14
ATP11A	rs76533333	13	113352916	A	G	0.92	-0.027	0.003	2.81E-29
ATP11A	rs1278769	13	113536627	A	G	0.24	-0.009	0.002	5.52E-12
GAS6	rs7994900	13	114553134	T	C	0.27	0.011	0.002	6.65E-14
SPTB	rs2273475	14	65268605	A	G	0.90	-0.013	0.002	1.99E-09
PSEN1	rs10151436	14	73616095	A	T	0.89	0.013	0.002	3.85E-11
HACD3	rs452306	15	65822777	T	C	0.59	-0.010	0.001	5.51E-13
ITFG3	rs11248914	16	293562	T	C	0.66	0.011	0.001	1.42E-14
RMI2	rs11643024	16	11443183	A	G	0.31	0.008	0.002	7.98E-10
CCDC101	rs7190771	16	28590030	A	G	0.38	0.009	0.001	6.02E-11
CDH1	rs7198799	16	68818390	T	C	0.29	0.008	0.001	4.76E-09
PIEZO1	rs837763	16	88853729	T	C	0.56	0.018	0.001	5.20E-38
ERAL1	rs9914988	17	27183104	A	G	0.80	0.013	0.002	4.66E-17
TMC6	rs2748427	17	76121864	A	G	0.78	-0.031	0.002	9.82E-49
FN3KRP	rs9909940	17	80689036	T	C	0.31	0.032	0.001	1.43E-116
FN3K	rs62076520	17	80695406	A	G	0.37	-0.026	0.001	3.06E-82
TBCD	rs191734192	17	80778724	A	G	0.13	0.042	0.003	3.75E-66
C18orf25	rs28671200	18	43774444	T	G	0.68	0.009	0.002	1.56E-08
MYO9B	rs17533945	19	17257802	T	C	0.60	-0.013	0.001	1.62E-23
PDCD5	rs12978547	19	33037212	C	G	0.97	0.028	0.004	3.96E-12
PDCD5	rs10405535	19	33072085	A	G	0.30	0.012	0.002	6.47E-14
RBM38	rs737092	20	55990405	T	C	0.51	-0.007	0.001	7.57E-09

HbA1c, glycated hemoglobin; SNP, single nucleotide polymorphisms; CHR, chromosome; BP, physical position of SNP (base-pairs); A1, effect allele; A2, alternative allele; EAF: effect allele frequency; Beta, effect allele beta coefficient; SE, standard error.

ESM Table 6. Characteristics of genome-wide significant single-nucleotide polymorphisms associated with 2hGlu$_{adj}$BMI.

Nearest Gene	SNP	CHR	BP	A1	A2	EAF	Beta	SE	*p*-value
GCKR	rs1260326	2	27730940	T	C	0.40	0.049	0.008	5.93E-12
COBLL1	rs12692738	2	165558252	T	C	0.76	0.049	0.009	2.72E-08
ADCY5	rs11708067	3	123065778	A	G	0.78	0.087	0.009	1.98E-22
CAMK2B	rs878521	7	44255643	A	G	0.24	0.099	0.009	1.25E-28
ABO	rs550057	9	136146597	T	C	0.28	0.053	0.009	3.62E-11
TCF7L2	rs7903146	10	114758349	T	C	0.26	0.085	0.009	2.79E-26
ABCC8	rs4148646	11	17415190	C	G	0.40	0.040	0.008	4.39E-08
HNF1A-AS1	rs2649999	12	121380544	T	C	0.36	0.050	0.008	2.01E-10
CLEC14A	rs112824462	14	38842759	A	G	0.25	-0.058	0.010	6.73E-09
VPS13C	rs17271305	15	62332980	A	G	0.58	-0.059	0.008	2.88E-14
SLC2A4	rs117643180	17	7185779	A	C	0.03	0.234	0.033	7.31E-14
GIPR	rs1800437	19	46181392	C	G	0.22	0.100	0.010	4.79E-26

2hGlu$_{adj}$BMI, 2h glucose adjusted for BMI; SNP, single nucleotide polymorphisms; CHR, chromosome; BP, physical position of SNP (base-pairs); A1, effect allele; A2, alternative allele; EAF: effect allele frequency; Beta, effect allele beta coefficient; SE, standard error.

ESM Table 7. Characteristics of genome-wide significant single-nucleotide polymorphisms associated with PCOS.

Nearest Gene	SNP	CHR	BP	A1	A2	EAF	Beta	SE	*p*-value
THADA	rs7563201	2	43561780	A	G	0.45	-0.108	0.017	3.68E-10
ERRB4	rs2178575	2	213391766	A	G	0.15	0.166	0.022	3.34E-14
IRF1/RAD50	rs13164856	5	131813204	T	C	0.73	0.124	0.019	1.45E-10
GATA4/NEIL2	rs804279	8	11623889	A	T	0.26	0.128	0.018	3.76E-12
PLGRKT	rs10739076	9	5440589	A	C	0.31	0.110	0.020	2.51E-08
FANCC	rs7864171	9	97723266	A	G	0.43	-0.093	0.017	2.95E-08
DENND1A	rs9696009	9	126619233	A	G	0.07	0.202	0.031	7.96E-11
ARL14EP/FSHB	rs11031005	11	30226356	T	C	0.85	-0.159	0.022	8.66E-13
YAP1	rs11225154	11	102043240	A	G	0.09	0.179	0.027	5.44E-11
ZBTB16	rs1784692	11	113949232	T	C	0.82	0.144	0.023	1.88E-10
ERRB3/RAB5B	rs2271194	12	56477694	A	T	0.42	0.097	0.017	4.57E-09
KRR1	rs1795379	12	75941042	T	C	0.24	-0.117	0.020	1.81E-09
TOX3	rs8043701	16	52375777	A	T	0.82	-0.127	0.021	9.61E-10
MAPRE1	rs853854	20	31420757	A	T	0.5	-0.098	0.016	2.36E-09

PCOS, polycystic ovary syndrome; SNP, single nucleotide polymorphisms; CHR, chromosome; BP, physical position of SNP (base-pairs); A1, effect allele; A2, alternative allele; EAF: effect allele frequency; Beta, effect allele beta coefficient; SE, standard error.

ESM Table 8. GWAS data sources used in the current study, number of IV, variance explained, and F-statistics.

Trait	Author	PMID	Source	Total samples or cases/controls	Unit	#IV	R2	F-statistics	Imputation reference panel	Ancestry	Link
T2DM	Anubha Mahajan	30297969	DIAGRAM	74,124 cases/824,006 controls	log OR	231	0.02	93.7	HRC and a population-specific reference panel of Icelandic	European	http://diagram-consortium.org/index.html
T2DMadjBMI				50,409 cases/523,897 controls	log OR	150	0.02	89.1			
FGadjBMI	Ji Chen	24509480	MAGIC	~200,000 individuals	mmol/l	85	0.036	87.8	1000 Genome	European	https://magicinvestigators.org/
FIadjBMI					pmol/l	42	0.006	28.7			
HbA1c					%	86	0.05	122.3			
2hGluadjBMI					mmol/l	12	0.008	132.4			
PCOS	Felix Day	30566500		4,790 cases/20,405 controls	log OR	14	0.005	40.8	Hapmap2 and 1000 Genome	European	https://www.repository.cam.ac.uk/handle/1810/283491

F = ((N-K-1)/K)(R^2/(1-R^2)), N denotes the sample size, K denotes the number of IVs. R^2 for T2DM and glycaemic traits were extracted from the original GWAS meta-analysis; for PCOS, R^2 was estimated using minor allele frequency. IV: instrumental variables.; PCOS, polycystic ovary syndrome; T2DM, type 2 diabetes mellitus; $T2DM_{adj}BMI$, type 2 diabetes adjusted for BMI; $FG_{adj}BMI$, fasting glucose adjusted for BMI; $FI_{adj}BMI$, fasting insulin adjusted for BMI; HbA1c, glycated haemoglobin A; $2hGlu_{adj}BMI$, 2h glucose after an oral glucose challenge adjusted for BMI. For PCOS GWAS data, 23andMe were not included due to limited availability.

ESM Table 9. Local genetic correlation between T2DM, glycaemic traits and PCOS with suggestive significance.

chr	start	end	num_snp	k	local_rhog	var	se	z	*p*
T2DM									
6	97842284	100630146	4008	50	4.56E-04	5.35E-08	2.31E-04	1.97	4.87E-02
10	80876749	82414679	3177	50	-6.52E-04	8.41E-08	2.90E-04	-2.25	2.46E-02
12	119754110	122007651	3672	50	7.24E-04	8.59E-08	2.93E-04	2.47	1.35E-02
16	53382572	55903774	4811	50	9.77E-04	1.14E-07	3.38E-04	2.89	3.84E-03
18	57630483	59020751	2063	50	8.93E-04	7.01E-08	2.65E-04	3.37	7.46E-04
$T2DM_{adj}BMI$									
3	11019665	13070799	3395	50	7.78E-04	1.13E-07	3.36E-04	2.32	2.05E-02
6	33236497	35455756	3393	50	5.92E-04	8.87E-08	2.98E-04	1.99	4.68E-02
10	80876749	82414679	3178	50	-6.87E-04	1.12E-07	3.35E-04	-2.05	4.02E-02
11	15742552	17578402	2553	50	6.20E-04	9.90E-08	3.15E-04	1.97	4.88E-02
12	119754110	122007651	3672	50	6.79E-04	1.01E-07	3.17E-04	2.14	3.23E-02
18	57630483	59020751	2063	50	4.97E-04	6.31E-08	2.51E-04	1.98	4.80E-02
$FG_{adj}BMI$									
4	103221356	105305294	3021	50	6.57E-04	1.04E-07	3.23E-04	2.03	4.19E-02
HbA1c									
10	37790029	42372579	2944	38	1.37E-03	2.50E-07	5.00E-04	2.75	5.99E-03

chr, chromosome; start, start position of the locus; end: end position of the locus; num_snp, number of SNPs in the locus; k, number of eigenvectors used in the truncated-SVD; local_rhog, local genetic covariance estimates; var, variance estimates; se, standard error estimate. PCOS, polycystic ovary syndrome; T2DM, type 2 diabetes mellitus; $T2DM_{adj}BMI$, type 2 diabetes adjusted for BMI; $FG_{adj}BMI$, fasting glucose adjusted for BMI; HbA1c, glycated hemoglobin A.

ESM Table 10. Detailed annotation of genome-wide significant SNPs identified by cross-trait meta-analysis.

SNP	Location	Consequence	IMPACT	SYMBOL	Feature_type	BIOTYPE
T2DM and PCOS						
rs72753599	1:214007176-214007176	intron_variant	MODIFIER	PROX1	Transcript	protein_coding
rs72753599	1:214007176-214007176	intron_variant	MODIFIER	PROX1	Transcript	protein_coding
rs72753599	1:214007176-214007176	intron_variant	MODIFIER	PROX1	Transcript	protein_coding
rs72753599	1:214007176-214007176	intron_variant	MODIFIER	PROX1	Transcript	protein_coding
rs8050136	16:53782363-53782363	intron_variant,NMD_transcript_variant	MODIFIER	FTO	Transcript	nonsense_mediated_decay
rs8050136	16:53782363-53782363	intron_variant	MODIFIER	FTO	Transcript	protein_coding
rs8050136	16:53782363-53782363	intron_variant,non_coding_transcript_variant	MODIFIER	FTO	Transcript	processed_transcript
rs8050136	16:53782363-53782363	intron_variant,non_coding_transcript_variant	MODIFIER	FTO	Transcript	processed_transcript
rs8050136	16:53782363-53782363	intron_variant	MODIFIER	FTO	Transcript	protein_coding
rs8050136	16:53782363-53782363	intron_variant	MODIFIER	FTO	Transcript	protein_coding
rs8050136	16:53782363-53782363	intron_variant,NMD_transcript_variant	MODIFIER	FTO	Transcript	nonsense_mediated_decay
rs8050136	16:53782363-53782363	intron_variant	MODIFIER	FTO	Transcript	protein_coding
rs8050136	16:53782363-53782363	intron_variant,NMD_transcript_variant	MODIFIER	FTO	Transcript	nonsense_mediated_decay
rs8050136	16:53782363-53782363	intron_variant,NMD_transcript_variant	MODIFIER	FTO	Transcript	nonsense_mediated_decay
rs8050136	16:53782363-53782363	intron_variant	MODIFIER	FTO	Transcript	protein_coding
rs8050136	16:53782363-53782363	intron_variant	MODIFIER	FTO	Transcript	protein_coding
rs8050136	16:53782363-53782363	regulatory_region_variant	MODIFIER	-	RegulatoryFeature	promoter_flanking_region
rs9675376	18:60302011-60302011	downstream_gene_variant	MODIFIER	-	Transcript	lncRNA
rs9675376	18:60302011-60302011	downstream_gene_variant	MODIFIER	-	Transcript	lncRNA
rs10938398	4:45184122-45184122	intergenic_variant	MODIFIER	-	-	-
$T2DM_{adj}BMI$ and PCOS						
rs72753599	1:214007176-214007176	intron_variant	MODIFIER	PROX1	Transcript	protein_coding
rs72753599	1:214007176-214007176	intron_variant	MODIFIER	PROX1	Transcript	protein_coding
rs72753599	1:214007176-214007176	intron_variant	MODIFIER	PROX1	Transcript	protein_coding
rs72753599	1:214007176-214007176	intron_variant	MODIFIER	PROX1	Transcript	protein_coding
rs9930501	16:53796540-53796540	intron_variant,NMD_transcript_variant	MODIFIER	FTO	Transcript	nonsense_mediated_decay
rs9930501	16:53796540-53796540	intron_variant	MODIFIER	FTO	Transcript	protein_coding
rs9930501	16:53796540-53796540	intron_variant,non_coding_transcript_variant	MODIFIER	FTO	Transcript	processed_transcript
rs9930501	16:53796540-53796540	intron_variant,non_coding_transcript_variant	MODIFIER	FTO	Transcript	processed_transcript
rs9930501	16:53796540-53796540	intron_variant	MODIFIER	FTO	Transcript	protein_coding
rs9930501	16:53796540-53796540	intron_variant	MODIFIER	FTO	Transcript	protein_coding
rs9930501	16:53796540-53796540	intron_variant,NMD_transcript_variant	MODIFIER	FTO	Transcript	nonsense_mediated_decay
rs9930501	16:53796540-53796540	intron_variant	MODIFIER	FTO	Transcript	protein_coding
rs9930501	16:53796540-53796540	intron_variant,NMD_transcript_variant	MODIFIER	FTO	Transcript	nonsense_mediated_decay
rs9930501	16:53796540-53796540	intron_variant,NMD_transcript_variant	MODIFIER	FTO	Transcript	nonsense_mediated_decay
rs9930501	16:53796540-53796540	intron_variant	MODIFIER	FTO	Transcript	protein_coding
rs9930501	16:53796540-53796540	intron_variant	MODIFIER	FTO	Transcript	protein_coding

rs2238689	19:45675403-45675403	intron_variant	MODIFIER	GIPR	Transcript	protein_coding
rs2238689	19:45675403-45675403	intron_variant	MODIFIER	GIPR	Transcript	protein_coding
rs2238689	19:45675403-45675403	intron_variant	MODIFIER	GIPR	Transcript	protein_coding
rs2238689	19:45675403-45675403	intron_variant	MODIFIER	GIPR	Transcript	protein_coding
rs2238689	19:45675403-45675403	intron_variant	MODIFIER	GIPR	Transcript	protein_coding
rs2238689	19:45675403-45675403	intron_variant	MODIFIER	GIPR	Transcript	protein_coding
rs2238689	19:45675403-45675403	downstream_gene_variant	MODIFIER	MIR642A	Transcript	miRNA
rs2238689	19:45675403-45675403	downstream_gene_variant	MODIFIER	MIR642A	Transcript	miRNA
rs2238689	19:45675403-45675403	downstream_gene_variant	MODIFIER	MIR642A	Transcript	miRNA
rs2238689	19:45675403-45675403	intron_variant,NMD_transcript_variant	MODIFIER	GIPR	Transcript	nonsense_mediated_decay
rs2238689	19:45675403-45675403	intron_variant,NMD_transcript_variant	MODIFIER	GIPR	Transcript	nonsense_mediated_decay
rs2238689	19:45675403-45675403	intron_variant,NMD_transcript_variant	MODIFIER	GIPR	Transcript	nonsense_mediated_decay
rs2238689	19:45675403-45675403	non_coding_transcript_exon_variant	MODIFIER	GIPR	Transcript	retained_intron
rs2238689	19:45675403-45675403	non_coding_transcript_exon_variant	MODIFIER	GIPR	Transcript	retained_intron
rs2238689	19:45675403-45675403	non_coding_transcript_exon_variant	MODIFIER	GIPR	Transcript	retained_intron
rs2238689	19:45675403-45675403	intron_variant	MODIFIER	GIPR	Transcript	protein_coding
rs2238689	19:45675403-45675403	intron_variant	MODIFIER	GIPR	Transcript	protein_coding
rs2238689	19:45675403-45675403	intron_variant	MODIFIER	GIPR	Transcript	protein_coding
rs2238689	19:45675403-45675403	upstream_gene_variant	MODIFIER	GIPR	Transcript	retained_intron
rs2238689	19:45675403-45675403	upstream_gene_variant	MODIFIER	GIPR	Transcript	retained_intron
rs2238689	19:45675403-45675403	upstream_gene_variant	MODIFIER	GIPR	Transcript	retained_intron
rs2238689	19:45675403-45675403	downstream_gene_variant	MODIFIER	GIPR	Transcript	protein_coding
rs2238689	19:45675403-45675403	downstream_gene_variant	MODIFIER	GIPR	Transcript	protein_coding
rs2238689	19:45675403-45675403	downstream_gene_variant	MODIFIER	GIPR	Transcript	protein_coding
rs2238689	19:45675403-45675403	upstream_gene_variant	MODIFIER	GIPR	Transcript	processed_transcript
rs2238689	19:45675403-45675403	upstream_gene_variant	MODIFIER	GIPR	Transcript	processed_transcript
rs2238689	19:45675403-45675403	upstream_gene_variant	MODIFIER	GIPR	Transcript	processed_transcript
rs2238689	19:45675403-45675403	upstream_gene_variant	MODIFIER	MIR642B	Transcript	miRNA
rs2238689	19:45675403-45675403	upstream_gene_variant	MODIFIER	MIR642B	Transcript	miRNA
rs2238689	19:45675403-45675403	upstream_gene_variant	MODIFIER	MIR642B	Transcript	miRNA
rs2238689	19:45675403-45675403	intron_variant	MODIFIER	GIPR	Transcript	protein_coding
rs2238689	19:45675403-45675403	intron_variant	MODIFIER	GIPR	Transcript	protein_coding
rs2238689	19:45675403-45675403	intron_variant	MODIFIER	GIPR	Transcript	protein_coding
rs1509097	2:164881421-164881421	intron_variant,non_coding_transcript_variant	MODIFIER	-	Transcript	lncRNA
rs1509097	2:164881421-164881421	intron_variant,non_coding_transcript_variant	MODIFIER	-	Transcript	lncRNA
rs1509097	2:164881421-164881421	intron_variant,non_coding_transcript_variant	MODIFIER	-	Transcript	lncRNA
rs1509097	2:164881421-164881421	intron_variant,non_coding_transcript_variant	MODIFIER	-	Transcript	lncRNA
rs1509097	2:164881421-164881421	intron_variant,non_coding_transcript_variant	MODIFIER	-	Transcript	lncRNA
rs3934729	3:123300916-123300916	intron_variant	MODIFIER	ADCY5	Transcript	protein_coding
rs3934729	3:123300916-123300916	intron_variant	MODIFIER	ADCY5	Transcript	protein_coding

rs3934729	3:123300916-123300916	intron_variant	MODIFIER	ADCY5	Transcript	protein_coding
rs3934729	3:123300916-123300916	intron_variant	MODIFIER	ADCY5	Transcript	protein_coding
rs3934729	3:123300916-123300916	intron_variant	MODIFIER	ADCY5	Transcript	protein_coding
rs3934729	3:123300916-123300916	intron_variant	MODIFIER	ADCY5	Transcript	protein_coding
rs3934729	3:123300916-123300916	intron_variant,non_coding_transcript_variant	MODIFIER	ADCY5	Transcript	retained_intron
rs3934729	3:123300916-123300916	intron_variant,non_coding_transcript_variant	MODIFIER	ADCY5	Transcript	retained_intron
rs3934729	3:123300916-123300916	intron_variant,non_coding_transcript_variant	MODIFIER	ADCY5	Transcript	retained_intron
rs3934729	3:123300916-123300916	intron_variant,non_coding_transcript_variant	MODIFIER	ADCY5	Transcript	retained_intron
rs3934729	3:123300916-123300916	intron_variant	MODIFIER	ADCY5	Transcript	protein_coding
rs3934729	3:123300916-123300916	intron_variant	MODIFIER	ADCY5	Transcript	protein_coding
rs3934729	3:123300916-123300916	regulatory_region_variant	MODIFIER	-	RegulatoryFeature	CTCF_binding_site
rs3934729	3:123300916-123300916	regulatory_region_variant	MODIFIER	-	RegulatoryFeature	CTCF_binding_site
FG_{adj}BMI and PCOS						
rs72753599	1:214007176-214007176	intron_variant	MODIFIER	PROX1	Transcript	protein_coding
rs72753599	1:214007176-214007176	intron_variant	MODIFIER	PROX1	Transcript	protein_coding
rs72753599	1:214007176-214007176	intron_variant	MODIFIER	PROX1	Transcript	protein_coding
rs72753599	1:214007176-214007176	intron_variant	MODIFIER	PROX1	Transcript	protein_coding
rs6485690	11:46777081-46777081	intron_variant	MODIFIER	CKAP5	Transcript	protein_coding
rs6485690	11:46777081-46777081	intron_variant	MODIFIER	CKAP5	Transcript	protein_coding
rs6485690	11:46777081-46777081	intron_variant	MODIFIER	CKAP5	Transcript	protein_coding
rs9844212	3:123303023-123303023	intron_variant	MODIFIER	ADCY5	Transcript	protein_coding
rs9844212	3:123303023-123303023	intron_variant	MODIFIER	ADCY5	Transcript	protein_coding
rs9844212	3:123303023-123303023	intron_variant	MODIFIER	ADCY5	Transcript	protein_coding
rs9844212	3:123303023-123303023	intron_variant,non_coding_transcript_variant	MODIFIER	ADCY5	Transcript	retained_intron
rs9844212	3:123303023-123303023	intron_variant,non_coding_transcript_variant	MODIFIER	ADCY5	Transcript	retained_intron
rs9844212	3:123303023-123303023	intron_variant	MODIFIER	ADCY5	Transcript	protein_coding
FI_{adj}BMI and PCOS						
rs3813583	16:79721183-79721183	intron_variant,non_coding_transcript_variant	MODIFIER	LINC01229	Transcript	lncRNA
rs3813583	16:79721183-79721183	intron_variant,non_coding_transcript_variant	MODIFIER	LINC01229	Transcript	lncRNA
rs3813583	16:79721183-79721183	intron_variant,non_coding_transcript_variant	MODIFIER	MAFTRR	Transcript	lncRNA
rs3813583	16:79721183-79721183	intron_variant,non_coding_transcript_variant	MODIFIER	MAFTRR	Transcript	lncRNA
rs3813583	16:79721183-79721183	intron_variant,non_coding_transcript_variant	MODIFIER	LINC01229	Transcript	lncRNA
rs3813583	16:79721183-79721183	intron_variant,non_coding_transcript_variant	MODIFIER	LINC01229	Transcript	lncRNA
rs3813583	16:79721183-79721183	downstream_gene_variant	MODIFIER	MAFTRR	Transcript	lncRNA
rs3813583	16:79721183-79721183	downstream_gene_variant	MODIFIER	MAFTRR	Transcript	lncRNA
rs3813583	16:79721183-79721183	intron_variant,non_coding_transcript_variant	MODIFIER	LINC01229	Transcript	lncRNA
rs3813583	16:79721183-79721183	intron_variant,non_coding_transcript_variant	MODIFIER	LINC01229	Transcript	lncRNA
rs3813583	16:79721183-79721183	intron_variant,non_coding_transcript_variant	MODIFIER	LINC01229	Transcript	lncRNA
rs3813583	16:79721183-79721183	intron_variant,non_coding_transcript_variant	MODIFIER	LINC01229	Transcript	lncRNA
rs3813583	16:79721183-79721183	intron_variant,non_coding_transcript_variant	MODIFIER	LINC01229	Transcript	lncRNA

rs3813583	16:79721183-79721183	intron_variant,non_coding_transcript_variant	MODIFIER	LINC01229	Transcript	lncRNA
rs3813583	16:79721183-79721183	intron_variant,non_coding_transcript_variant	MODIFIER	MAFTRR	Transcript	lncRNA
rs3813583	16:79721183-79721183	intron_variant,non_coding_transcript_variant	MODIFIER	MAFTRR	Transcript	lncRNA
rs3813583	16:79721183-79721183	downstream_gene_variant	MODIFIER	MAFTRR	Transcript	lncRNA
rs3813583	16:79721183-79721183	downstream_gene_variant	MODIFIER	MAFTRR	Transcript	lncRNA
rs3813583	16:79721183-79721183	intron_variant,non_coding_transcript_variant	MODIFIER	LINC01229	Transcript	lncRNA
rs3813583	16:79721183-79721183	intron_variant,non_coding_transcript_variant	MODIFIER	LINC01229	Transcript	lncRNA
rs3813583	16:79721183-79721183	intron_variant,non_coding_transcript_variant	MODIFIER	LINC01229	Transcript	lncRNA
rs3813583	16:79721183-79721183	intron_variant,non_coding_transcript_variant	MODIFIER	LINC01229	Transcript	lncRNA
rs3813583	16:79721183-79721183	intron_variant,non_coding_transcript_variant	MODIFIER	LINC01229	Transcript	lncRNA
rs3813583	16:79721183-79721183	intron_variant,non_coding_transcript_variant	MODIFIER	LINC01229	Transcript	lncRNA
rs3813583	16:79721183-79721183	intron_variant,non_coding_transcript_variant	MODIFIER	MAFTRR	Transcript	lncRNA
rs3813583	16:79721183-79721183	intron_variant,non_coding_transcript_variant	MODIFIER	MAFTRR	Transcript	lncRNA
rs3813583	16:79721183-79721183	intron_variant,non_coding_transcript_variant	MODIFIER	LINC01229	Transcript	lncRNA
rs3813583	16:79721183-79721183	intron_variant,non_coding_transcript_variant	MODIFIER	LINC01229	Transcript	lncRNA
rs3813583	16:79721183-79721183	intron_variant,non_coding_transcript_variant	MODIFIER	LINC01229	Transcript	lncRNA
rs3813583	16:79721183-79721183	intron_variant,non_coding_transcript_variant	MODIFIER	LINC01229	Transcript	lncRNA
rs3813583	16:79721183-79721183	intron_variant,non_coding_transcript_variant	MODIFIER	LINC01229	Transcript	lncRNA
rs3813583	16:79721183-79721183	intron_variant,non_coding_transcript_variant	MODIFIER	LINC01229	Transcript	lncRNA
rs3813583	16:79721183-79721183	intron_variant,non_coding_transcript_variant	MODIFIER	LINC01229	Transcript	lncRNA
rs3813583	16:79721183-79721183	intron_variant,non_coding_transcript_variant	MODIFIER	LINC01229	Transcript	lncRNA
rs3813583	16:79721183-79721183	intron_variant,non_coding_transcript_variant	MODIFIER	LINC01229	Transcript	lncRNA
rs3813583	16:79721183-79721183	intron_variant,non_coding_transcript_variant	MODIFIER	LINC01229	Transcript	lncRNA
rs3813583	16:79721183-79721183	upstream_gene_variant	MODIFIER	LINC01229	Transcript	lncRNA
rs3813583	16:79721183-79721183	upstream_gene_variant	MODIFIER	LINC01229	Transcript	lncRNA
rs3813583	16:79721183-79721183	intron_variant,non_coding_transcript_variant	MODIFIER	LINC01229	Transcript	lncRNA
rs3813583	16:79721183-79721183	intron_variant,non_coding_transcript_variant	MODIFIER	LINC01229	Transcript	lncRNA
rs3813583	16:79721183-79721183	upstream_gene_variant	MODIFIER	LINC01229	Transcript	lncRNA
rs3813583	16:79721183-79721183	upstream_gene_variant	MODIFIER	LINC01229	Transcript	lncRNA
rs3813583	16:79721183-79721183	intron_variant,non_coding_transcript_variant	MODIFIER	LINC01229	Transcript	lncRNA
rs3813583	16:79721183-79721183	intron_variant,non_coding_transcript_variant	MODIFIER	LINC01229	Transcript	lncRNA
rs3813583	16:79721183-79721183	intron_variant,non_coding_transcript_variant	MODIFIER	LINC01229	Transcript	lncRNA
rs3813583	16:79721183-79721183	intron_variant,non_coding_transcript_variant	MODIFIER	LINC01229	Transcript	lncRNA
rs3813583	16:79721183-79721183	intron_variant,non_coding_transcript_variant	MODIFIER	LINC01229	Transcript	lncRNA
rs3813583	16:79721183-79721183	intron_variant,non_coding_transcript_variant	MODIFIER	LINC01229	Transcript	lncRNA
rs3813583	16:79721183-79721183	upstream_gene_variant	MODIFIER	LINC01229	Transcript	lncRNA
rs3813583	16:79721183-79721183	upstream_gene_variant	MODIFIER	LINC01229	Transcript	lncRNA
rs3813583	16:79721183-79721183	intron_variant,non_coding_transcript_variant	MODIFIER	LINC01229	Transcript	lncRNA
rs3813583	16:79721183-79721183	intron_variant,non_coding_transcript_variant	MODIFIER	LINC01229	Transcript	lncRNA
rs3813583	16:79721183-79721183	intron_variant,non_coding_transcript_variant	MODIFIER	LINC01229	Transcript	lncRNA

rs3813583	16:79721183-79721183	intron_variant,non_coding_transcript_variant	MODIFIER	LINC01229	Transcript	lncRNA
rs3813583	16:79721183-79721183	intron_variant,non_coding_transcript_variant	MODIFIER	LINC01229	Transcript	lncRNA
rs3813583	16:79721183-79721183	intron_variant,non_coding_transcript_variant	MODIFIER	LINC01229	Transcript	lncRNA
rs3813583	16:79721183-79721183	intron_variant,non_coding_transcript_variant	MODIFIER	LINC01229	Transcript	lncRNA
rs3813583	16:79721183-79721183	intron_variant,non_coding_transcript_variant	MODIFIER	LINC01229	Transcript	lncRNA
rs3813583	16:79721183-79721183	intron_variant,non_coding_transcript_variant	MODIFIER	MAFTRR	Transcript	lncRNA
rs3813583	16:79721183-79721183	intron_variant,non_coding_transcript_variant	MODIFIER	MAFTRR	Transcript	lncRNA
rs3813583	16:79721183-79721183	downstream_gene_variant	MODIFIER	MAFTRR	Transcript	lncRNA
rs3813583	16:79721183-79721183	downstream_gene_variant	MODIFIER	MAFTRR	Transcript	lncRNA
rs3813583	16:79721183-79721183	intron_variant,non_coding_transcript_variant	MODIFIER	LINC01229	Transcript	lncRNA
rs3813583	16:79721183-79721183	intron_variant,non_coding_transcript_variant	MODIFIER	LINC01229	Transcript	lncRNA
rs3813583	16:79721183-79721183	intron_variant,non_coding_transcript_variant	MODIFIER	LINC01229	Transcript	lncRNA
rs3813583	16:79721183-79721183	intron_variant,non_coding_transcript_variant	MODIFIER	LINC01229	Transcript	lncRNA
rs3813583	16:79721183-79721183	intron_variant,non_coding_transcript_variant	MODIFIER	LINC01229	Transcript	lncRNA
rs3813583	16:79721183-79721183	intron_variant,non_coding_transcript_variant	MODIFIER	LINC01229	Transcript	lncRNA
rs3813583	16:79721183-79721183	intron_variant,non_coding_transcript_variant	MODIFIER	LINC01229	Transcript	lncRNA
rs3813583	16:79721183-79721183	intron_variant,non_coding_transcript_variant	MODIFIER	LINC01229	Transcript	lncRNA
rs3813583	16:79721183-79721183	intron_variant,non_coding_transcript_variant	MODIFIER	LINC01229	Transcript	lncRNA
rs3813583	16:79721183-79721183	intron_variant,non_coding_transcript_variant	MODIFIER	LINC01229	Transcript	lncRNA
rs3813583	16:79721183-79721183	downstream_gene_variant	MODIFIER	MAFTRR	Transcript	lncRNA
rs3813583	16:79721183-79721183	downstream_gene_variant	MODIFIER	MAFTRR	Transcript	lncRNA
rs3813583	16:79721183-79721183	regulatory_region_variant	MODIFIER	-	RegulatoryFeature	promoter_flanking_region
rs3813583	16:79721183-79721183	regulatory_region_variant	MODIFIER	-	RegulatoryFeature	promoter_flanking_region
rs4135247	3:12355089-12355089	intron_variant	MODIFIER	PPARG	Transcript	protein_coding
rs4135247	3:12355089-12355089	intron_variant	MODIFIER	PPARG	Transcript	protein_coding
rs4135247	3:12355089-12355089	intron_variant	MODIFIER	PPARG	Transcript	protein_coding
rs4135247	3:12355089-12355089	intron_variant	MODIFIER	PPARG	Transcript	protein_coding
rs4135247	3:12355089-12355089	intron_variant	MODIFIER	PPARG	Transcript	protein_coding
rs4135247	3:12355089-12355089	intron_variant	MODIFIER	PPARG	Transcript	protein_coding
rs4135247	3:12355089-12355089	intron_variant	MODIFIER	PPARG	Transcript	protein_coding
rs4135247	3:12355089-12355089	intron_variant	MODIFIER	PPARG	Transcript	protein_coding
rs4135247	3:12355089-12355089	intron_variant	MODIFIER	PPARG	Transcript	protein_coding
rs4135247	3:12355089-12355089	intron_variant	MODIFIER	PPARG	Transcript	protein_coding
rs4135247	3:12355089-12355089	intron_variant,NMD_transcript_variant	MODIFIER	PPARG	Transcript	nonsense_mediated_decay
rs4135247	3:12355089-12355089	intron_variant,NMD_transcript_variant	MODIFIER	PPARG	Transcript	nonsense_mediated_decay
rs4135247	3:12355089-12355089	intron_variant	MODIFIER	PPARG	Transcript	protein_coding
rs4135247	3:12355089-12355089	intron_variant	MODIFIER	PPARG	Transcript	protein_coding
rs4135247	3:12355089-12355089	intron_variant	MODIFIER	PPARG	Transcript	protein_coding
rs4135247	3:12355089-12355089	intron_variant	MODIFIER	PPARG	Transcript	protein_coding
rs4135247	3:12355089-12355089	intron_variant	MODIFIER	PPARG	Transcript	protein_coding

rs4135247	3:12355089-12355089	intron_variant	MODIFIER	PPARG	Transcript	protein_coding
rs4135247	3:12355089-12355089	intron_variant	MODIFIER	PPARG	Transcript	protein_coding
rs4135247	3:12355089-12355089	intron_variant	MODIFIER	PPARG	Transcript	protein_coding
rs4135247	3:12355089-12355089	intron_variant,non_coding_transcript_variant	MODIFIER	PPARG	Transcript	retained_intron
rs4135247	3:12355089-12355089	intron_variant,non_coding_transcript_variant	MODIFIER	PPARG	Transcript	retained_intron
rs4135247	3:12355089-12355089	intron_variant,non_coding_transcript_variant	MODIFIER	PPARG	Transcript	retained_intron
rs4135247	3:12355089-12355089	intron_variant,non_coding_transcript_variant	MODIFIER	PPARG	Transcript	retained_intron
rs4135247	3:12355089-12355089	intron_variant	MODIFIER	PPARG	Transcript	protein_coding
rs4135247	3:12355089-12355089	intron_variant	MODIFIER	PPARG	Transcript	protein_coding
rs4135247	3:12355089-12355089	intron_variant	MODIFIER	PPARG	Transcript	protein_coding
rs4135247	3:12355089-12355089	intron_variant	MODIFIER	PPARG	Transcript	protein_coding
rs4135247	3:12355089-12355089	intron_variant	MODIFIER	PPARG	Transcript	protein_coding
rs4135247	3:12355089-12355089	intron_variant	MODIFIER	PPARG	Transcript	protein_coding
rs4135247	3:12355089-12355089	intron_variant	MODIFIER	PPARG	Transcript	protein_coding
rs4135247	3:12355089-12355089	intron_variant	MODIFIER	PPARG	Transcript	protein_coding
rs4135247	3:12355089-12355089	intron_variant	MODIFIER	PPARG	Transcript	protein_coding
rs4135247	3:12355089-12355089	intron_variant	MODIFIER	PPARG	Transcript	protein_coding
rs4135247	3:12355089-12355089	intron_variant	MODIFIER	PPARG	Transcript	protein_coding
rs4135247	3:12355089-12355089	intron_variant	MODIFIER	PPARG	Transcript	protein_coding
rs4135247	3:12355089-12355089	intron_variant	MODIFIER	PPARG	Transcript	protein_coding
rs4135247	3:12355089-12355089	intron_variant	MODIFIER	PPARG	Transcript	protein_coding
rs4135247	3:12355089-12355089	intron_variant,NMD_transcript_variant	MODIFIER	PPARG	Transcript	nonsense_mediated_decay
rs4135247	3:12355089-12355089	intron_variant,NMD_transcript_variant	MODIFIER	PPARG	Transcript	nonsense_mediated_decay
rs4135247	3:12355089-12355089	intron_variant	MODIFIER	PPARG	Transcript	protein_coding
rs4135247	3:12355089-12355089	intron_variant	MODIFIER	PPARG	Transcript	protein_coding
rs4135247	3:12355089-12355089	intron_variant	MODIFIER	PPARG	Transcript	protein_coding
rs4135247	3:12355089-12355089	intron_variant	MODIFIER	PPARG	Transcript	protein_coding
rs4135247	3:12355089-12355089	intron_variant	MODIFIER	PPARG	Transcript	protein_coding
rs4135247	3:12355089-12355089	intron_variant	MODIFIER	PPARG	Transcript	protein_coding
rs4135247	3:12355089-12355089	intron_variant	MODIFIER	PPARG	Transcript	protein_coding
rs4135247	3:12355089-12355089	intron_variant	MODIFIER	PPARG	Transcript	protein_coding
rs4135247	3:12355089-12355089	intron_variant	MODIFIER	PPARG	Transcript	protein_coding
rs4135247	3:12355089-12355089	intron_variant	MODIFIER	PPARG	Transcript	protein_coding
rs4135247	3:12355089-12355089	intron_variant,NMD_transcript_variant	MODIFIER	PPARG	Transcript	nonsense_mediated_decay
rs4135247	3:12355089-12355089	intron_variant,NMD_transcript_variant	MODIFIER	PPARG	Transcript	nonsense_mediated_decay
rs745379	8:11758186-11758186	intron_variant	MODIFIER	GATA4	Transcript	protein_coding
rs745379	8:11758186-11758186	intron_variant,non_coding_transcript_variant	MODIFIER	GATA4	Transcript	retained_intron
rs745379	8:11758186-11758186	downstream_gene_variant	MODIFIER	GATA4	Transcript	protein_coding
rs745379	8:11758186-11758186	intron_variant	MODIFIER	GATA4	Transcript	protein_coding
rs745379	8:11758186-11758186	intron_variant	MODIFIER	GATA4	Transcript	protein_coding

rs745379	8:11758186-11758186	intron_variant	MODIFIER	GATA4	Transcript	protein_coding
rs745379	8:11758186-11758186	regulatory_region_variant	MODIFIER	-	RegulatoryFeature	promoter_flanking_region
rs745379	CHR_HG76_PATCH:8194627-8194627	downstream_gene_variant	MODIFIER	GATA4	Transcript	protein_coding
rs745379	CHR_HG76_PATCH:8194627-8194627	intron_variant	MODIFIER	GATA4	Transcript	protein_coding
rs745379	CHR_HG76_PATCH:8194627-8194627	upstream_gene_variant	MODIFIER	-	Transcript	protein_coding
rs745379	CHR_HG76_PATCH:8194627-8194627	intron_variant	MODIFIER	GATA4	Transcript	protein_coding
rs745379	CHR_HG76_PATCH:8194627-8194627	intron_variant	MODIFIER	GATA4	Transcript	protein_coding
rs745379	CHR_HG76_PATCH:8194627-8194627	intron_variant,non_coding_transcript_variant	MODIFIER	GATA4	Transcript	retained_intron
rs745379	CHR_HG76_PATCH:8194627-8194627	intron_variant	MODIFIER	GATA4	Transcript	protein_coding
rs745379	CHR_HG76_PATCH:8194627-8194627	upstream_gene_variant	MODIFIER	-	Transcript	protein_coding
HbA1c and PCOS						
rs1265564	12:111270654-111270654	intron_variant	MODIFIER	CUX2	Transcript	protein_coding
rs1265564	12:111270654-111270654	intron_variant	MODIFIER	CUX2	Transcript	protein_coding
rs1265564	12:111270654-111270654	non_coding_transcript_exon_variant	MODIFIER	CUX2	Transcript	processed_transcript
rs1265564	12:111270654-111270654	regulatory_region_variant	MODIFIER	-	RegulatoryFeature	promoter_flanking_region
rs8047587	16:53764710-53764710	intron_variant,NMD_transcript_variant	MODIFIER	FTO	Transcript	nonsense_mediated_decay
rs8047587	16:53764710-53764710	intron_variant	MODIFIER	FTO	Transcript	protein_coding
rs8047587	16:53764710-53764710	intron_variant,non_coding_transcript_variant	MODIFIER	FTO	Transcript	processed_transcript
rs8047587	16:53764710-53764710	intron_variant,non_coding_transcript_variant	MODIFIER	FTO	Transcript	processed_transcript
rs8047587	16:53764710-53764710	intron_variant	MODIFIER	FTO	Transcript	protein_coding
rs8047587	16:53764710-53764710	intron_variant	MODIFIER	FTO	Transcript	protein_coding
rs8047587	16:53764710-53764710	intron_variant,NMD_transcript_variant	MODIFIER	FTO	Transcript	nonsense_mediated_decay
rs8047587	16:53764710-53764710	intron_variant	MODIFIER	FTO	Transcript	protein_coding
rs8047587	16:53764710-53764710	intron_variant,NMD_transcript_variant	MODIFIER	FTO	Transcript	nonsense_mediated_decay
rs8047587	16:53764710-53764710	intron_variant,NMD_transcript_variant	MODIFIER	FTO	Transcript	nonsense_mediated_decay
rs8047587	16:53764710-53764710	intron_variant	MODIFIER	FTO	Transcript	protein_coding
rs8047587	16:53764710-53764710	intron_variant	MODIFIER	FTO	Transcript	protein_coding
rs2238689	19:45675403-45675403	intron_variant	MODIFIER	GIPR	Transcript	protein_coding
rs2238689	19:45675403-45675403	intron_variant	MODIFIER	GIPR	Transcript	protein_coding
rs2238689	19:45675403-45675403	intron_variant	MODIFIER	GIPR	Transcript	protein_coding
rs2238689	19:45675403-45675403	intron_variant	MODIFIER	GIPR	Transcript	protein_coding
rs2238689	19:45675403-45675403	intron_variant	MODIFIER	GIPR	Transcript	protein_coding
rs2238689	19:45675403-45675403	intron_variant	MODIFIER	GIPR	Transcript	protein_coding
rs2238689	19:45675403-45675403	downstream_gene_variant	MODIFIER	MIR642A	Transcript	miRNA
rs2238689	19:45675403-45675403	downstream_gene_variant	MODIFIER	MIR642A	Transcript	miRNA
rs2238689	19:45675403-45675403	downstream_gene_variant	MODIFIER	MIR642A	Transcript	miRNA
rs2238689	19:45675403-45675403	intron_variant,NMD_transcript_variant	MODIFIER	GIPR	Transcript	nonsense_mediated_decay
rs2238689	19:45675403-45675403	intron_variant,NMD_transcript_variant	MODIFIER	GIPR	Transcript	nonsense_mediated_decay
rs2238689	19:45675403-45675403	intron_variant,NMD_transcript_variant	MODIFIER	GIPR	Transcript	nonsense_mediated_decay
rs2238689	19:45675403-45675403	non_coding_transcript_exon_variant	MODIFIER	GIPR	Transcript	retained_intron

rs2238689	19:45675403-45675403	non_coding_transcript_exon_variant	MODIFIER	GIPR	Transcript	retained_intron
rs2238689	19:45675403-45675403	non_coding_transcript_exon_variant	MODIFIER	GIPR	Transcript	retained_intron
rs2238689	19:45675403-45675403	intron_variant	MODIFIER	GIPR	Transcript	protein_coding
rs2238689	19:45675403-45675403	intron_variant	MODIFIER	GIPR	Transcript	protein_coding
rs2238689	19:45675403-45675403	intron_variant	MODIFIER	GIPR	Transcript	protein_coding
rs2238689	19:45675403-45675403	upstream_gene_variant	MODIFIER	GIPR	Transcript	retained_intron
rs2238689	19:45675403-45675403	upstream_gene_variant	MODIFIER	GIPR	Transcript	retained_intron
rs2238689	19:45675403-45675403	upstream_gene_variant	MODIFIER	GIPR	Transcript	retained_intron
rs2238689	19:45675403-45675403	downstream_gene_variant	MODIFIER	GIPR	Transcript	protein_coding
rs2238689	19:45675403-45675403	downstream_gene_variant	MODIFIER	GIPR	Transcript	protein_coding
rs2238689	19:45675403-45675403	downstream_gene_variant	MODIFIER	GIPR	Transcript	protein_coding
rs2238689	19:45675403-45675403	upstream_gene_variant	MODIFIER	GIPR	Transcript	processed_transcript
rs2238689	19:45675403-45675403	upstream_gene_variant	MODIFIER	GIPR	Transcript	processed_transcript
rs2238689	19:45675403-45675403	upstream_gene_variant	MODIFIER	GIPR	Transcript	processed_transcript
rs2238689	19:45675403-45675403	upstream_gene_variant	MODIFIER	MIR642B	Transcript	miRNA
rs2238689	19:45675403-45675403	upstream_gene_variant	MODIFIER	MIR642B	Transcript	miRNA
rs2238689	19:45675403-45675403	upstream_gene_variant	MODIFIER	MIR642B	Transcript	miRNA
rs2238689	19:45675403-45675403	intron_variant	MODIFIER	GIPR	Transcript	protein_coding
rs2238689	19:45675403-45675403	intron_variant	MODIFIER	GIPR	Transcript	protein_coding
rs2238689	19:45675403-45675403	intron_variant	MODIFIER	GIPR	Transcript	protein_coding
rs4731113	7:123643895-123643895	downstream_gene_variant	MODIFIER	ASB15	Transcript	protein_coding
rs4731113	7:123643895-123643895	downstream_gene_variant	MODIFIER	ASB15	Transcript	protein_coding

SNP, single nucleotide polymorphisms; T2DM, type 2 diabetes mellitus; PCOS, polycystic ovary syndrome; $T2DM_{adj}BMI$, type 2 diabetes adjusted for BMI; $FG_{adj}BMI$, fasting glucose adjusted for BMI; $FI_{adj}BMI$, fasting insulin adjusted for BMI; HbA1c, glycated hemoglobin A.

ESM Table 11. TWAS significant genes for PCOS across 48 GTEx tissues (version 7).

ID	CHR	Tissue	BEST.GWAS.ID	BEST.GWAS.Z	TWAS.Z	TWAS.*p*	FDR
ARL14EP	11	Artery Aorta	rs10835649	5.00	4.75	2.00E-06	1.28E-02
ARL14EP	11	Artery Tibial	rs10835649	5.00	4.36	1.29E-05	4.99E-02
ARL14EP	11	Testis	rs10835649	5.00	5.24	1.63E-07	1.49E-03
ARL14EP	11	Thyroid	rs10835649	5.00	4.71	2.48E-06	2.42E-02
RP11-345M22.2	16	Adrenal Gland	rs17767383	4.41	-4.38	1.21E-05	3.94E-02
RP11-345M22.2	16	Artery Tibial	rs17767383	4.41	-4.41	1.05E-05	4.99E-02
RP11-345M22.2	16	Brain Frontal Cortex BA9	rs17767383	4.41	-4.38	1.21E-05	3.50E-02
RP11-345M22.2	16	Brain Spinal cord cervical c-1	rs17767383	4.41	-4.41	1.03E-05	2.05E-02
RP11-345M22.2	16	Colon Transverse	rs17767383	4.41	-4.45	8.62E-06	4.53E-02
RP11-345M22.2	16	Esophagus Muscularis	rs17767383	4.41	-4.57	4.99E-06	3.84E-02
RP11-345M22.2	16	Liver	rs17767383	4.41	-4.38	1.21E-05	3.49E-02
RP11-345M22.2	16	Ovary	rs17767383	4.41	-4.33	1.48E-05	4.10E-02
RP11-345M22.2	16	Prostate	rs17767383	4.41	-4.38	1.21E-05	3.35E-02
RP11-345M22.2	16	Small Intestine Terminal Ileum	rs17767383	4.41	-4.32	1.53E-05	4.35E-02
RP11-345M22.2	16	Stomach	rs17767383	4.41	-4.38	1.16E-05	4.48E-02
RP11-345M22.2	16	Thyroid	rs17767383	4.41	-4.37	1.27E-05	4.13E-02
RP11-736K20.6	11	Artery Tibial	rs6592325	-4.36	-4.21	2.55E-05	4.99E-02
RP11-736K20.6	11	Stomach	rs6592325	-4.36	-4.21	2.55E-05	4.48E-02
SERPINB8	18	Stomach	rs2162352	3.71	-4.17	3.05E-05	4.48E-02
SNORD3B-2	17	Artery Tibial	rs11652784	3.75	-4.25	2.13E-05	4.99E-02
UBL3	13	Thyroid	rs558107	3.75	4.47	7.85E-06	3.83E-02

TWAS, transcriptome-wide association studies; ID, gene identifier; CHR, chromosome; BEST.GWAS.ID, rsID of the most significant GWAS SNP in locus; BEST.GWAS.Z, Z-score of the most significant GWAS SNP in locus; TWAS.Z, TWAS Z-score; TWAS.*p*, TWAS *p*-value.

ESM Table 12. Estimates of odds ratio of genetically predicted T2DM, glycaemic traits on PCOS.

Traits	#IV	OR (95%CI)	*p*-value	*p* for pleiotropy
T2DM				
IVW	209	1.15(1.06, 1.25)	1.27×10^{-3}	
MR-Egger	209	1.10(0.93, 1.31)	0.26	0.43
SIMEX corrected MR-Egger	209	1.10(0.93, 1.32)	0.27	
Weighted median	209	1.09(0.96, 1.16)	0.17	
$T2DM_{adj}BMI$				
IVW	141	1.06(0.96, 1.16)	0.26	
MR-Egger	141	0.92(0.75, 1.12)	0.40	0.15
SIMEX corrected MR-Egger	141	0.93 (0.75, 1.14)	0.23	
Weighted median	141	0.95(0.82, 1.10)	0.51	
$FG_{adj}BMI$				
IVW	80	0.92(0.66, 1.28)	0.62	
MR-Egger	80	0.68(0.37, 1.24)	0.21	0.24
SIMEX corrected MR-Egger	80	0.90 (0.64, 1.26)	0.55	
Weighted median	80	0.95(0.60, 1.51)	0.84	
$FI_{adj}BMI$				
IVW	42	2.85(1.37, 5.92)	4.9×10^{-3}	
MR-Egger	42	1.02(0.12, 8.94)	0.99	0.33
SIMEX corrected MR-Egger	42	2.83 (1.31, 6.15)	0.01	
Weighted median	42	3.08(1.10, 8.59)	0.04	
HbA1c				
IVW	81	1.20(0.70, 2.07)	0.50	
MR-Egger	81	0.99(0.35, 2.81)	0.99	0.67
SIMEX corrected MR-Egger	81	1.24 (0.72, 2.15)	0.44	
Weighted median	81	0.89(0.41, 1.94)	0.77	
$2hGlu_{adj}BMI$				
IVW	12	0.76(0.49, 1.18)	0.22	
MR-Egger	12	0.35(0.12, 1.02)	0.08	0.15
SIMEX corrected MR-Egger	12	0.57 (0.82, 1.24)	0.06	
Weighted median	12	0.81(0.53, 1.23)	0.32	

IV, instrumental variable; T2DM, type 2 diabetes mellitus; $T2DM_{adj}BMI$, type 2 diabetes adjusted for BMI; $FG_{adj}BMI$, fasting glucose adjusted for BMI; $FI_{adj}BMI$, fasting insulin adjusted for BMI; HbA1c, glycated haemoglobin A; $2hGlu_{adj}BMI$, 2h glucose after an oral glucose challenge adjusted for BMI; IVW: inverse-variance weighted; SIMEX: simulation extrapolation.

ESM Table 13. I^2_{GX} estimates for the causal effect of each T2DM, glycaemic trait on PCOS.

Trait	I^2_{GX}
Type 2 diabetes mellitus	0.94
$T2DM_{adj}BMI$	0.93
$FG_{adj}BMI$	0.97
$FI_{adj}BMI$	0.73
HbA1c	0.95
$2hGlu_{adj}BMI$	0.76

T2DM, type 2 diabetes mellitus; $T2DM_{adj}BMI$, type 2 diabetes adjusted for BMI; $FG_{adj}BMI$, fasting glucose adjusted for BMI; $FI_{adj}BMI$, fasting insulin adjusted for BMI; HbA1c, glycated haemoglobin A; $2hGlu_{adj}BMI$, 2h glucose after an oral glucose challenge adjusted for BMI.

ESM Table 14. Between SNP heterogeneity statistics for the IVW analyses of causal effects of T2DM, glycaemic traits on PCOS.

Trait	Q	df	*p*
Type 2 diabetes mellitus	272.15	208	1.85E-03
$T2DM_{adj}BMI$	198.07	140	9.05E-04
$FG_{adj}BMI$	83.15	79	0.35
$FI_{adj}BMI$	48.16	41	0.20
HbA1c	93.65	80	0.14
$2hGlu_{adj}BMI$	26.28	11	5.80E-03

T2DM, type 2 diabetes mellitus; $T2DM_{adj}BMI$, type 2 diabetes adjusted for BMI; $FG_{adj}BMI$, fasting glucose adjusted for BMI; $FI_{adj}BMI$, fasting insulin adjusted for BMI; HbA1c, glycated haemoglobin A; $2hGlu_{adj}BMI$, 2h glucose after an oral glucose challenge adjusted for BMI.

ESM Table 15. Estimates of causal effect of genetically predicted T2DM, glycaemic traits on PCOS using MR-PRESSO.

Exposure	# of outliers	Methods	OR (95% CI)	*p*-value for association	*p*-value for global heterogeneity
T2D	1	raw	1.15 (1.06, 1.23)	1.47×10^{-3}	1.8×10^{-3}
		outliers-corrected	1.13 (1.04, 1.21)	5.61×10^{-3}	
$T2D_{adj}BMI$	2	raw	1.06 (0.96, 1.15)	0.26	6.0×10^{-4}
		outliers-corrected	1.06 (0.97, 1.14)	0.22	
$FG_{adj}BMI$	0	raw	0.92 (0.59, 1.25)	0.62	0.37
$FI_{adj}BMI$	0	raw	2.85 (2.12, 3.58)	7.5×10^{-3}	0.19
HbA1c	0	raw	1.20 (0.66, 1.75)	0.50	0.15
$2hGlu_{adj}BMI$	1	raw	0.76 (0.32, 1.20)	0.25	0.14
		outliers-corrected	0.89 (0.47, 1.31)	0.59	

ESM Table 16. Estimates of causal effect of genetically predicted T2DM, glycaemic traits on PCOS using Steiger filtering.

Exposure	Outcome	# of IVs removed	# of IVs remained	OR (95% CI)	*p*-value
T2D	PCOS	51	158	1.04 (0.98, 1.11)	0.18
$T2D_{adj}BMI$	PCOS	21	120	1.03 (0.96, 1.22)	0.32
$FG_{adj}BMI$	PCOS	3	77	0.97 (0.70, 1.33)	0.83
$FI_{adj}BMI$	PCOS	3	39	2.56 (1.27, 5.18)	0.01
HbA1c	PCOS	7	74	1.07 (0.69, 1.67)	0.75
$2hGlu_{adj}BMI$	PCOS	0	12	0.76 (0.49, 1.18)	0.22

ESM Table 17. Cross-trait meta-analysis between female T2DM and PCOS. SNPs with *p*-CPASSOC < 5E-8 and single trait *p*-value < 1E-3 are presented.

SNP	CHR	BP	A1	A2	EAF	Beta_PCOS	Beta_trait	p_{PCOS}	$p_{singlet\ rait}$	$p_{CPASSOC}$	Genes within clumping ranges
T2DM and PCOS											
rs9933509	16	53818167	T	C	0.57	-0.15	-0.13	3.10×10^{-06}	3.20×10^{-39}	1.68×10^{-45}	*FTO*
rs2550733	16	81449060	A	G	0.43	0.13	0.06	1.10×10^{-04}	6.60×10^{-09}	1.66×10^{-10}	
rs9378248	6	31326289	A	G	0.38	0.12	0.05	3.50×10^{-04}	3.80×10^{-07}	2.39×10^{-08}	
rs2238689	19	46178661	T	C	0.57	0.11	-0.05	5.40×10^{-04}	1.80×10^{-07}	2.51×10^{-09}	*GIPR, MIR642A, MIR642B*
rs340835	1	214163675	A	G	0.49	0.11	0.06	6.90×10^{-04}	1.30×10^{-08}	5.02×10^{-10}	*PROX1*
rs3934729	3	123019763	T	C	0.61	-0.11	0.05	7.40×10^{-04}	1.80×10^{-06}	4.42×10^{-08}	*ADCY5*
rs17773430	18	57963117	T	C	0.68	-0.11	-0.05	8.90×10^{-04}	7.00×10^{-07}	1.67×10^{-08}	

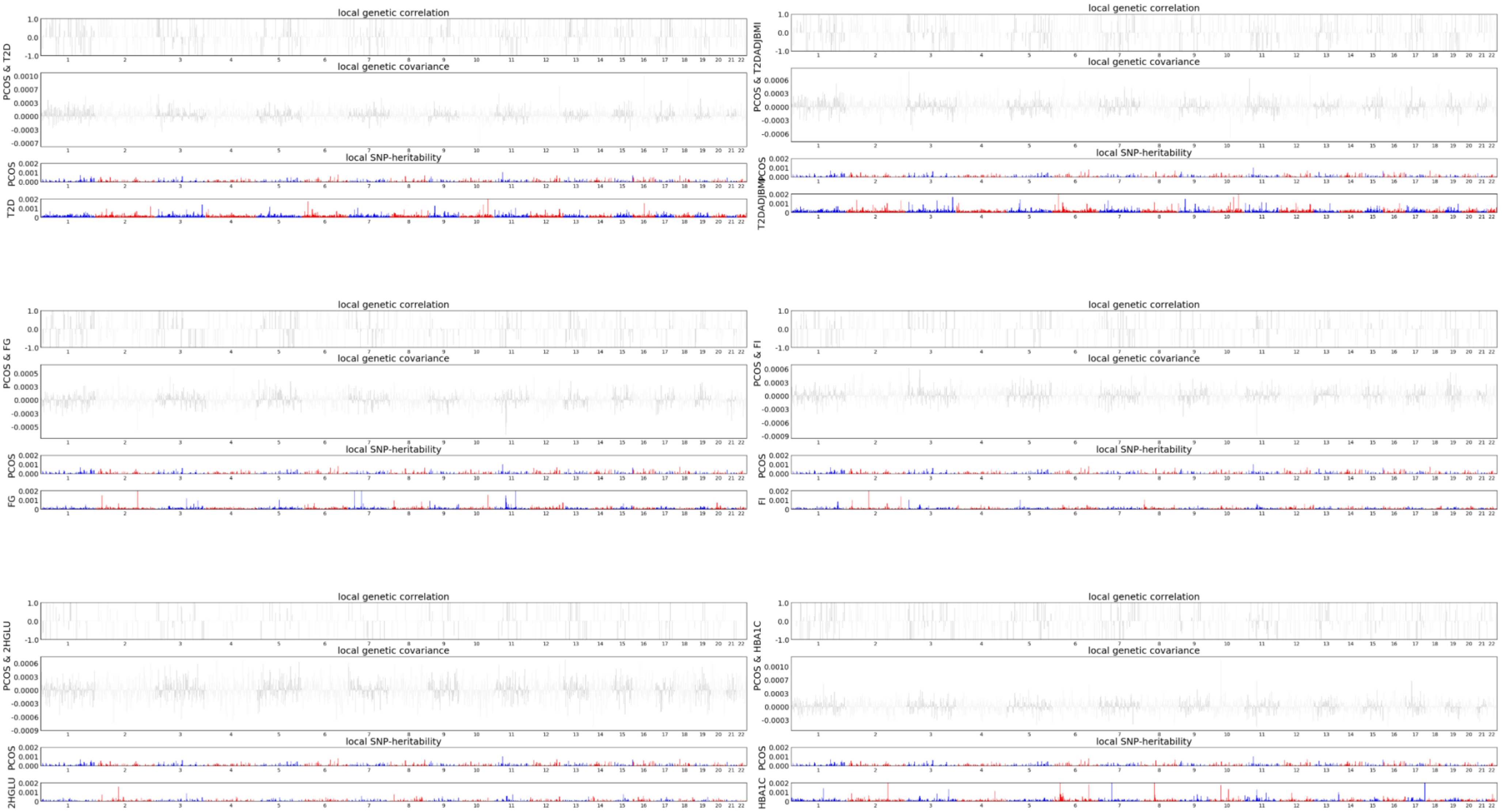

ESM Figure 1. **Local genetic correlation, genetic covariance and SNP heritability between T2DM, glycaemic traits and PCOS.** Coloured bars represent loci that show significant local genetic correlation and covariance after multiple testing adjustment. PCOS, polycystic ovary syndrome; T2DM, type 2 diabetes mellitus; T2DM$_{adj}$BMI, type 2 diabetes adjusted for BMI; FG$_{adj}$BMI, fasting glucose adjusted for BMI; FI$_{adj}$BMI, fasting insulin adjusted for BMI; HbA1c, glycated haemoglobin A; 2hGlu$_{adj}$BMI, 2h glucose after an oral glucose challenge adjusted for BMI.

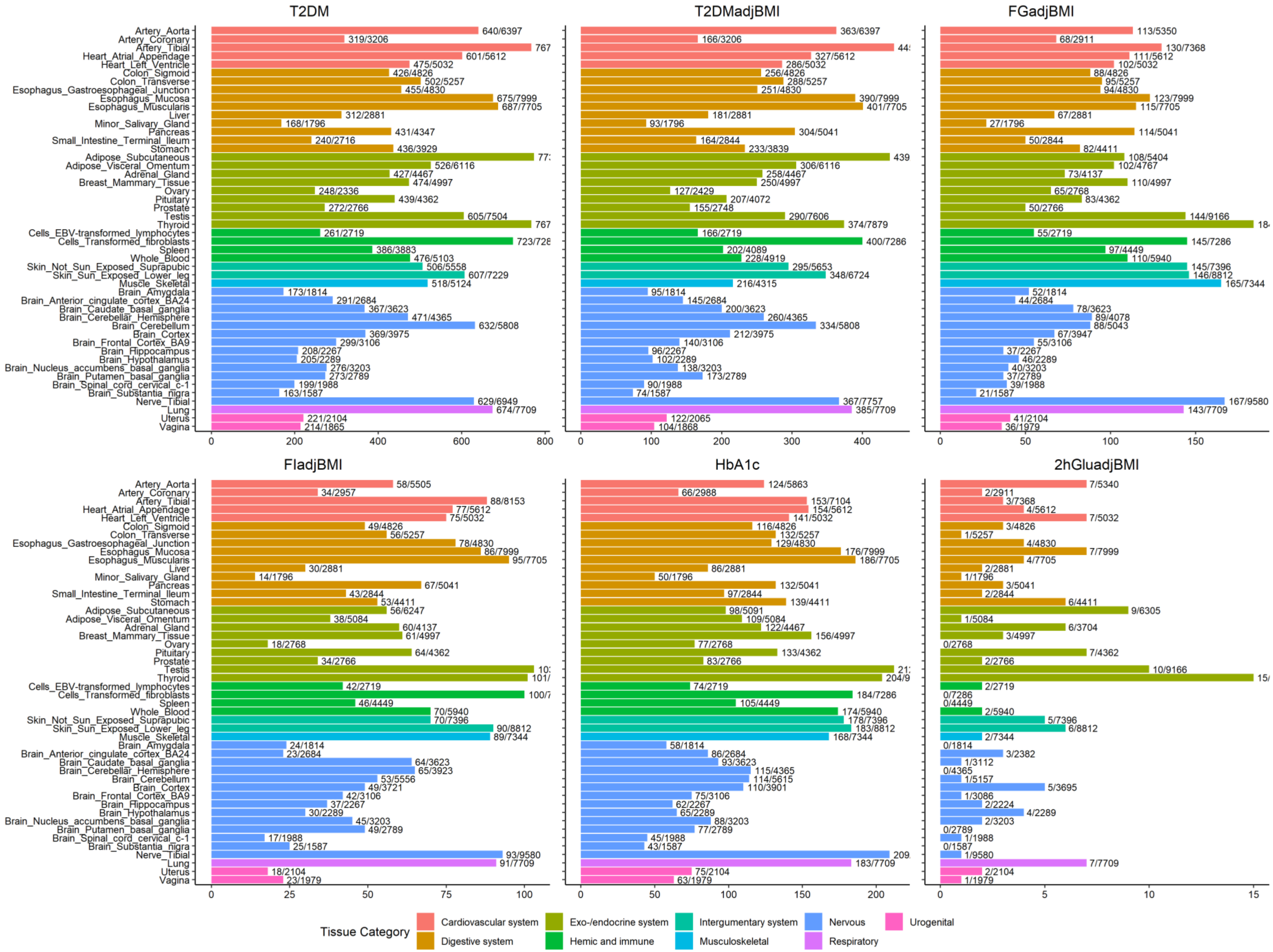

ESM Figure 2. Number of transcriptomic-wide association studies (TWAS) significant genes for T2DM, glycaemic traits across 48 GTEx tissues (version 7). T2DM, type 2 diabetes mellitus; PCOS, polycystic ovary syndrome; T2DM$_{adj}$BMI, type 2 diabetes adjusted for BMI; FG$_{adj}$BMI, fasting glucose adjusted for BMI; FI$_{adj}$BMI, fasting insulin adjusted for BMI; HbA1c, glycated haemoglobin A; 2hGlu$_{adj}$BMI, 2h glucose after an oral glucose challenge adjusted for BMI.

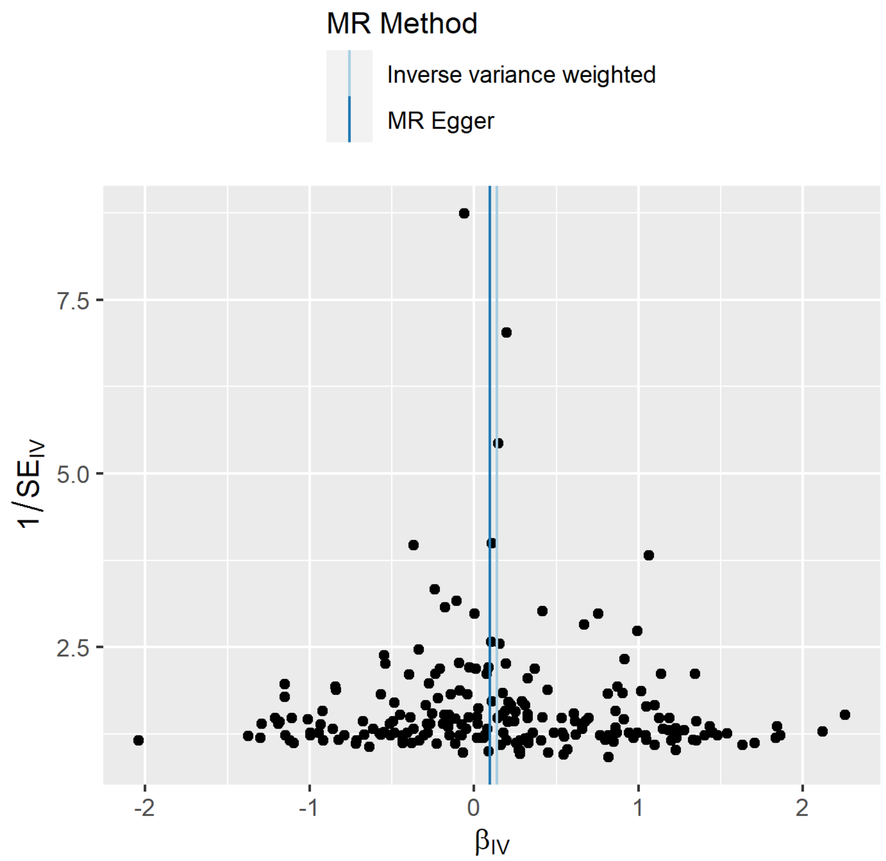

ESM Figure 3. Funnel plot assessing the extent to which pleiotropy is balanced across the set of instruments used in Mendelian randomization analysis of type 2 diabetes.

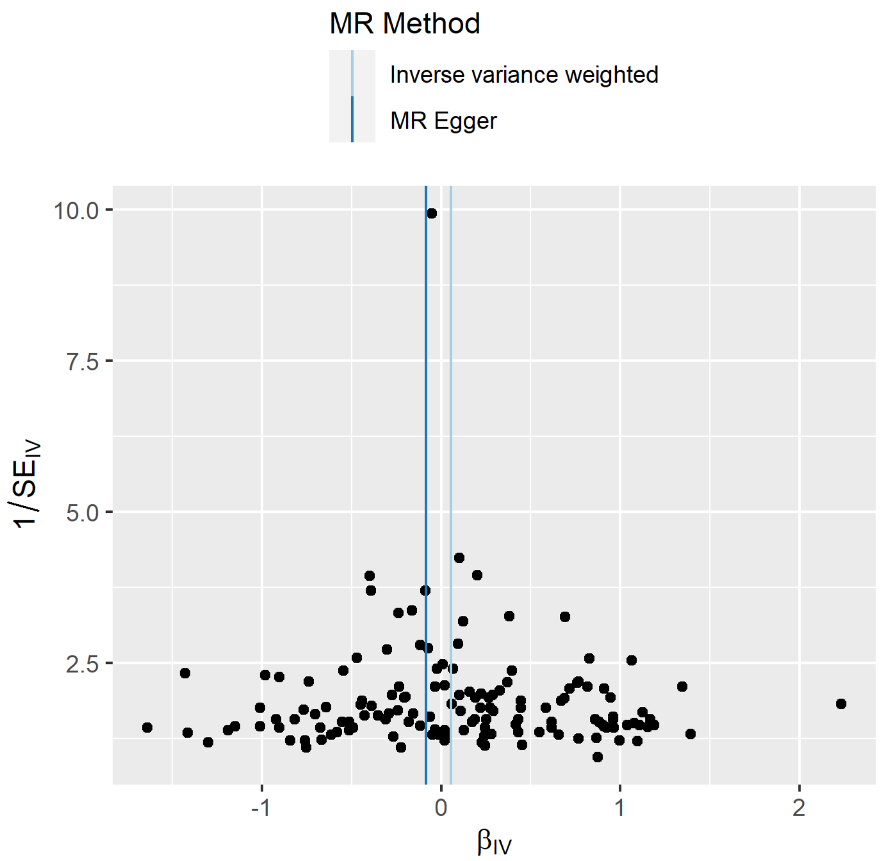

ESM Figure 4. Funnel plot assessing the extent to which pleiotropy is balanced across the set of instruments used in Mendelian randomization analysis of type 2 diabetes adjusted for BMI.

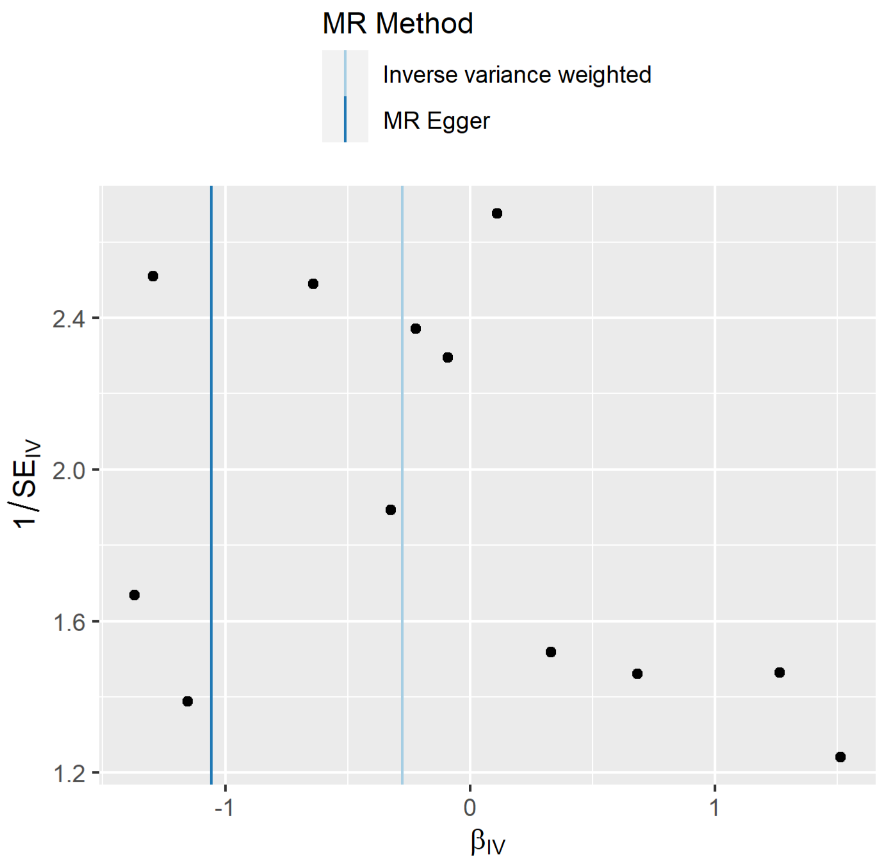

ESM Figure 5. **Funnel plot assessing the extent to which pleiotropy is balanced across the set of instruments used in Mendelian randomization analysis of 2h glucose challenge adjusted for BMI.**

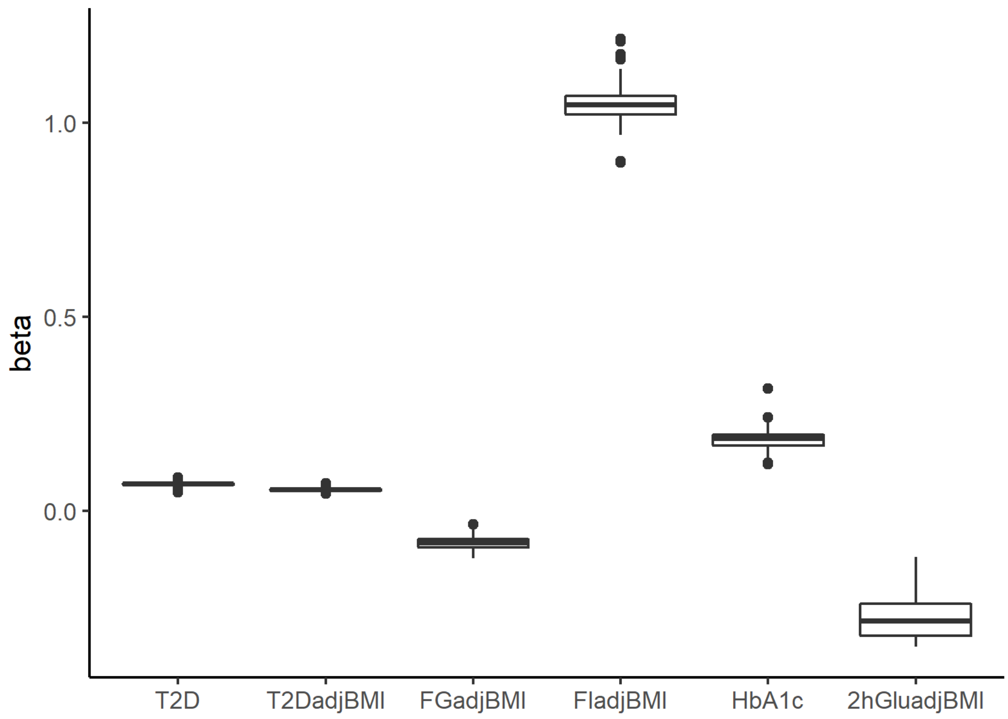

ESM Figure 6. Box plot of betas in leave-one-out analysis. beta: effect allele beta coefficient; T2DM, type 2 diabetes mellitus; $T2DM_{adj}BMI$, type 2 diabetes adjusted for BMI; $FG_{adj}BMI$, fasting glucose adjusted for BMI; $FI_{adj}BMI$, fasting insulin adjusted for BMI; HbA1c, glycated haemoglobin A; $2hGlu_{adj}BMI$, 2h glucose after an oral glucose challenge adjusted for BMI.

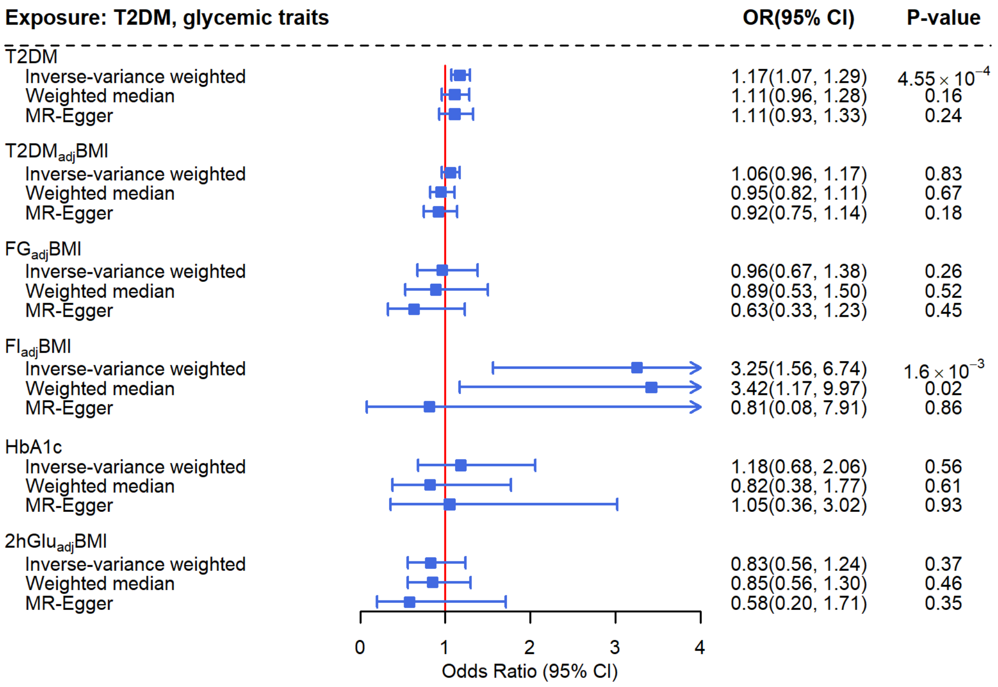

ESM Figure 7. Estimates of causal effect for genetically predicted T2DM, glycaemic traits and PCOS removing palindromic SNPs. The boxes denote the point estimate of causal effects, and the error bar denote 95% confidence intervals. Inverse-variance weighted approach was used as primary analysis, MR-Egger and weighted median approaches were used as sensitivity analysis. T2DM, type 2 diabetes mellitus; $T2DM_{adj}BMI$, type 2 diabetes adjusted for BMI; $FG_{adj}BMI$, fasting glucose adjusted for BMI; $FI_{adj}BMI$, fasting insulin adjusted for BMI; HbA1c, glycated hemoglobin A; $2hGlu_{adj}BMI$, 2h glucose after an oral glucose challenge adjusted for BMI.

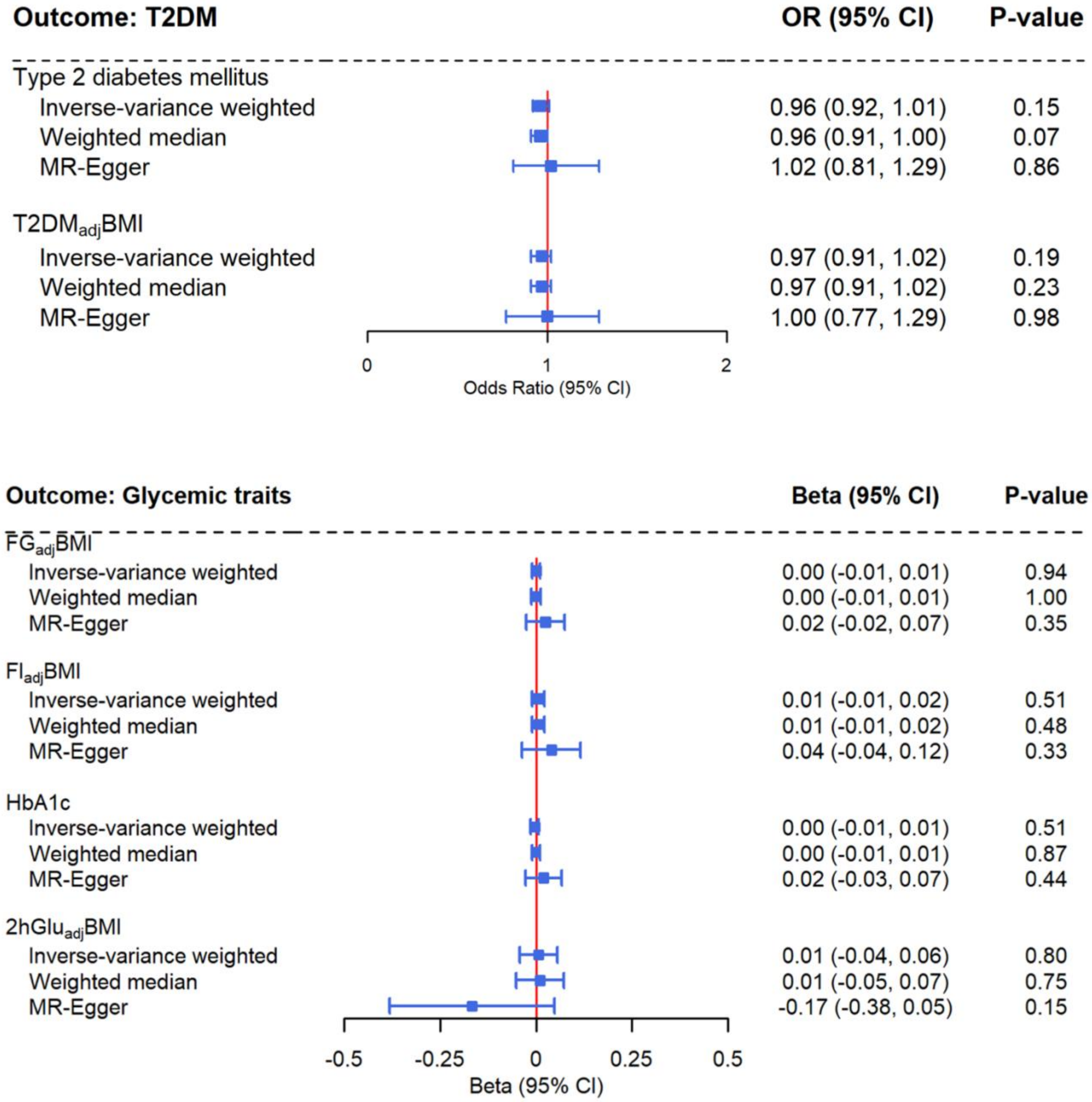

ESM Figure 8. Estimates of causal effect sizes for genetically predisposition to PCOS and T2DM, glycaemic traits using all genome-wide significant SNPs. The boxes denote the point estimate of causal effects, and the error bar denote 95% confidence intervals. Inverse-variance weighted approach was used as primary analysis, MR-Egger and weighted median approaches were used as sensitivity analysis. T2DM, type 2 diabetes mellitus; $T2DM_{adj}BMI$, type 2 diabetes adjusted for BMI. $FG_{adj}BMI$, fasting glucose adjusted for BMI; $FI_{adj}BMI$, fasting insulin adjusted for BMI; HbA1c, glycated haemoglobin A; $2hGlu_{adj}BMI$, 2h glucose after an oral glucose challenge adjusted for BMI.

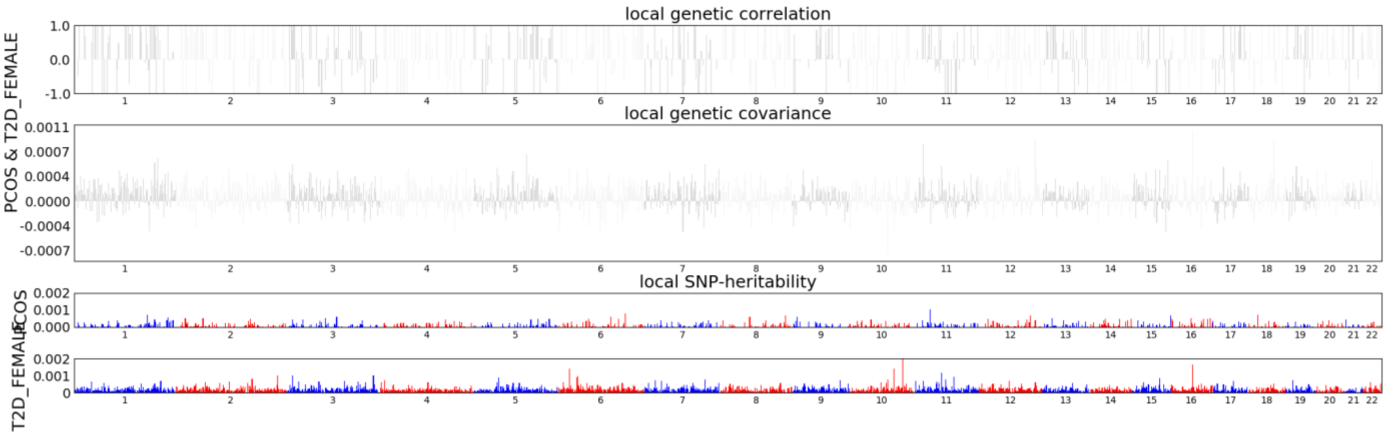

ESM Figure 9. **Local genetic correlation, genetic covariance and SNP heritability between female T2DM, glycaemic traits and PCOS.** Coloured bars represent loci that show significant local genetic correlation and covariance after multiple testing adjustment. PCOS, polycystic ovary syndrome; T2DM, type 2 diabetes mellitus.
